# Fragmentation of Dicarboxylic and Tricarboxylic Acids in the Krebs Cycle Using GC-EI-MS and GC-EI-MS/MS

**DOI:** 10.5702/massspectrometry.A0073

**Published:** 2019-08-30

**Authors:** Nobuyuki Okahashi, Shuichi Kawana, Junko Iida, Hiroshi Shimizu, Fumio Matsuda

**Affiliations:** 1 Department of Bioinformatic Engineering, Graduate School of Information Science and Technology, Osaka University, 1–5 Yamadaoka, Suita, Osaka, Japan; 2 Analytical and Measuring Instruments Division, Shimadzu Corporation, 1 Nishinokyo Kuwabara-cho, Nakagyo-ku, Kyoto, Japan; 3 Osaka University Shimadzu Analytical Innovation Research Laboratory, Graduate School of Engineering, Osaka University, 2–1 Yamadaoka, Suita, Osaka, Japan

**Keywords:** organic acids, fragmentation, GC-MS, electron ionization, collision-induced dissociation

## Abstract

Isotope labeling measurements using mass spectrometry can provide informative insights on the metabolic systems of various organisms. The detailed identification of carbon positions included in the fragment ions of dicarboxylic and tricarboxylic acids in central carbon metabolism is needed for precise interpretation of the metabolic states. In this study, fragment ions containing the carbon backbone cleavage of dicarboxylic and tricarboxylic in the Krebs cycle were investigated by using gas chromatography (GC)-electron ionization (EI)-MS and GC-EI-MS/MS. The positions of decarboxylation in the dicarboxylic and tricarboxylic acids were successfully identified by analyses using position-specific ^13^C-labeled standards prepared by *in vitro* enzymatic reactions. For example, carboxyl groups of C1 and C6 of trimethylsilyl (TMS)- and *tert*-butyldimethylsilyl (TBDMS)-derivatized malic and citric acids were primarily cleaved by EI. MS/MS analyses were also performed, and fragment ions of TBDMS-citric and α-ketoglutaric acids (αKG) with the loss of two carboxyl groups in collision-induced dissociation (CID) were observed.

## INTRODUCTION

Isotope labeling experiments using mass spectrometry (MS) are one of the powerful methods to study metabolic systems. In particular, ^13^C-labeling of dicarboxylic and tricarboxylic acids in the Krebs cycle is measured in the fields of biotechnology, systems biology, and medical sciences.^1–4)^ Since the interpretation of ^13^C-labeling data requires positional information about the carbon atoms in the fragment ions, identification of carbon atoms contained in the fragment ions as observed by MS and tandem-MS has been investigated.^5–10)^ However, the carbon positions in the decarboxylated fragment ions derived from dicarboxylic and tricarboxylic acids have not been experimentally validated, resulting in the loss of rich information. In this study, the electron ionization (EI)- and collision-induced dissociation (CID)-fragmentation of the abundant dicarboxylic and tricarboxylic acids in the Krebs cycle, *viz.* citric, αKG, succinic, fumaric, and malic acids was investigated to maximize the accessible data from a single analysis. The organic acid-derived carbon atoms included in the fragment ions were successfully identified by the analyses of position-specific ^13^C-labeled standards synthesized by *in vitro* enzymatic reactions.

## EXPERIMENTAL

### Chemicals

Non-labeled standards were purchased from Sigma-Aldrich (St. Louis, MO, USA). [1,2,3,4-^13^C]α-Ketoglutaric acid (99%), NaH^13^CO_3_ (99%), [1-^13^C]pyruvic acid (99%), [2-^13^C]pyruvic acid (99%), [3-^13^C]pyruvic acid (99%), and [1-^13^C]acetic acid (99%) were purchased from Cambridge Isotope Laboratories (Andover, MA, USA). Fully ^13^C-labeled organic acids were prepared from the extract of yeast cultured in a medium containing [U-^13^C]glucose as the sole carbon source following the previously described method.^11)^

### Preparation of position-specific ^13^C-labeled standards

*In vitro* enzymatic reaction was used for the synthesis of position-specific ^13^C-labeled standards as described previously with minor modification.^12)^ [4-^13^C]Malic acid was prepared *via* the reactions of phosphoenolpyruvate (PEP) carboxylase and malate dehydrogenase. Sixty-six millimolar *Tris*-HCl (pH=9), 10 mM PEP, 10 mM MgCl_2_, 20 mM NaH^13^CO_3_, 0.15 mM NADH, 4 units of PEP carboxylase from *Zea mais* leaves (Wako Pure Chemical Industries, Ltd. Corporation, Osaka, Japan), and 2 units of malate dehydrogenase from porcine heart (Wako Pure Chemical Industries, Ltd. Corporation) were gently mixed and incubated overnight at room temperature (Fig. S1a). [1-^13^C], [2-^13^C], and [3-^13^C]Malic acids were prepared *via* the sequential reactions of pyruvate kinase, PEP carboxylase, and malate dehydrogenase. Hundred millimolar *Tris*-HCl (pH=8), 10 mM ATP, 10 mM ^13^C-labeled pyruvic acid, 15 mM MgCl_2_, 20 mM NaHCO_3_, 10 mM NADH, 16 U of pyruvate kinase from rabbit muscle *ca.* 350 U/mg protein suspension (Wako Pure Chemical Industries, Ltd. Corporation), 4 U of PEP carboxylase from *Zea mais* leaves, and 2 U of malate dehydrogenase from porcine heart (Fig. S1a) were gently mixed and incubated overnight at room temperature. [1-^13^C], [2-^13^C], and [3-^13^C]Pyruvic acids were used for the synthesis of [1-^13^C], [2-^13^C], and [3-^13^C]malic acids, respectively. [1-^13^C], [5-^13^C], [6-^13^C], and [1,5-^13^C]Citric acids were prepared *via* the multiple reactions of pyruvate kinase, PEP carboxylase, acetyl-CoA synthetase (ACS), and citrate synthase (CS). Hundred millimolar *Tris*-HCl (pH=8), 10 mM pyruvic acid, 20 mM NaHCO_3_, 10 mM MgCl_2_, 10 mM ATP, 10 mM acetate, 10 mM CoA, 16 U of pyruvate kinase, 4 U of PEP carboxylase from *Zea mais* leaves, 2 U of citrate synthase, and 0.2 U of acetyl-CoA synthase were gently mixed and incubated overnight at room temperature. ACS and CS were obtained from the content of F-kit acetate (J.K. International, Tokyo, Japan) (Fig. S1b). Acetic acid, NaHCO_3_, and pyruvic acid were replaced with [1-^13^C]acetic acid, [1-^13^C]pyruvic acid, and NaH^13^CO_3_ for the synthesis of [1-^13^C], [5-^13^C], and [6-^13^C]citric acids, respectively.

### Derivatization of organic acids

Standard solution of organic acids was evaporated to dryness by Speed Vac (Thermo Fischer Scientific, Waltham, MA, USA). The dried standards were derivatized by adding 50 μL of 40 mg/mL methoxyamine hydrochloride pyridine solution and incubated for 1 h at 30°C. Subsequently 50 μL of *N*-methyl-*N*-trimethylsilyltrifluoroacetamide containing 1% 2,2,2-trifluoro-*N*-methyl-*N*-(trimethylsilyl)-acetamide, chlorotrimethylsilane (Thermo Fischer Scientific) or *N*-(*tert*-butyldimethylsilyl)-*N*-methyltrifluoroacetamide containing 1% *tert*-butyldimethylchlorosilane (Thermo Fischer Scientific) was added and the mixture was incubated for 1 h at 37 or 95°C for trimethylsilylation or *tert*-butyldimethylsilylation, respectively.^8)^ After 1 h of cooling, an aliquot of the supernatant was subjected to the analysis.

### GC-MS and GC-MS/MS analysis

GC-MS (GCMS-QP2010 Ultra, Shimadzu, Kyoto, Japan) and GC-MS/MS (GCMS-TQ8040, Shimadzu) equipped with DB-5MS+DG column ((30 m ×0.25 mm ID ×0.25 μm), Agilent Technologies, Santa Clara, CA, USA) were used.^8)^ Analysis conditions were as follows: constant flow rate of helium at 1.0 mL/min; ion source temperature, 230°C; electron impact ionization, 70 eV; injection volume, 1 μL; injection, pulsed split (split ratio, 1 : 10); oven temperature, 60°C for 3.5 min, increased at a rate of 10°C/min to 325°C, and maintained at that temperature for 10 min or 70°C for 2 min, increased at a rate of 3°C/min to 280°C, and maintained at that temperature for 5 min for the analysis of TMS- and TBDMS-derivatives, respectively. Argon (200 kPa) was used as collision gas for the MS/MS analysis. Collision energy was optimized using Smart MRM (Shimadzu, Kyoto, Japan). The obtained raw MS and MS/MS spectra were deposited in MassBank.^13)^

## RESULTS

### EI-induced fragmentation of TMS- and TBDMS-derivatized organic acids

**Figure 1:**
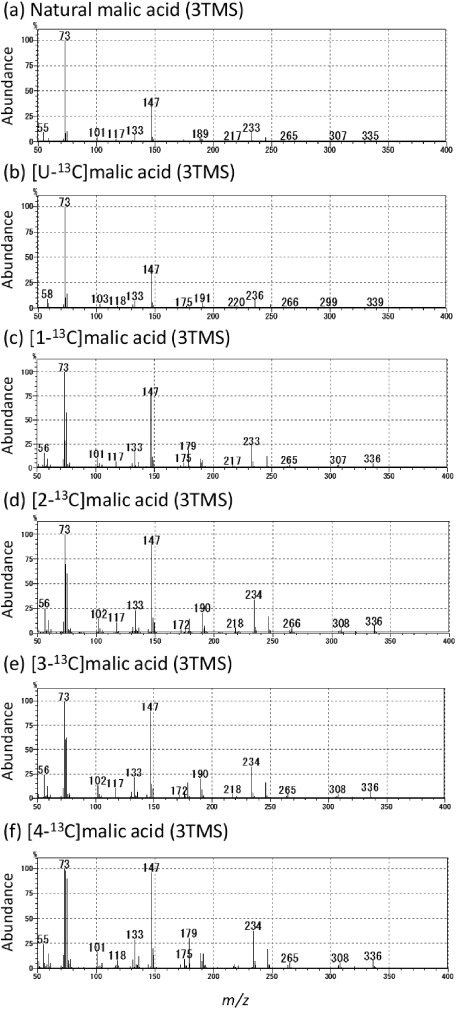
Fig. 1. EI-induced fragmentation of TMS-derivatized malic acid.

**Figure 2:**
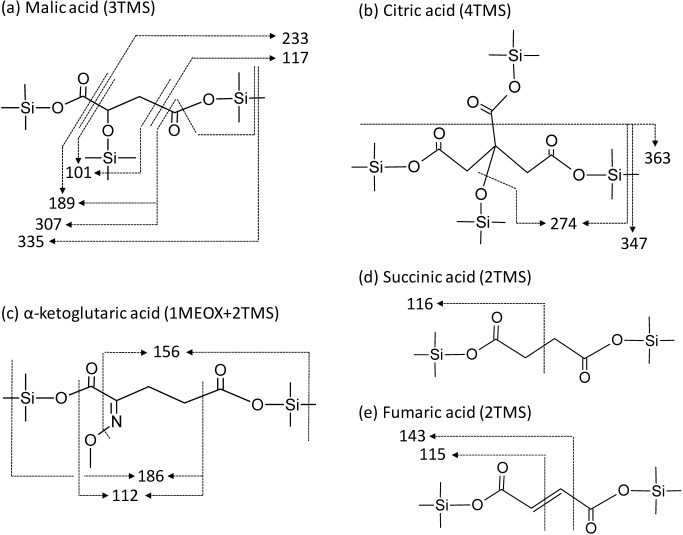
Fig. 2. Estimated EI-fragmentation of TMS-derivatized organic acids.

In this study, the EI- and CID-fragmentation of TMS- and TBDMS-derivatized dicarboxylic and tricarboxylic acids in the Krebs cycle was investigated by GC-EI-MS and GC-EI-MS/MS. To begin with, the TMS-derivatives of organic acids, one of the major analytes for metabolome analysis, were analyzed using GC-EI-MS. Figure 1 shows the EI-spectra of the TMS-derivatized malic acid. The ions with the highest intensity were 73 and 147, derived from TMS group.^14)^ The other commonly observed fragment ions in TMS-derivatives were [M−15]^+^ and [M−117]^+^, in which the fragments were cleaved at the methyl group in TMS group and TMS-carboxyl group. The EI-spectra of TMS-derivatized malic acid produced intense [M−15]^+^ (*m*/*z*=335) and [M−117]^+^ (*m*/*z*=233) ions with relatively minor ions (*m*/*z*=101, 117, 189, 217, 265, and 307). These data are consistent with the previous study.^14)^ To identify the carbon atoms included in these fragment ions, [U-^13^C]malic acid obtained from yeast cultured in [U-^13^C]glucose medium was derivatized and analyzed. As expected, the *m*/*z* of [M−15]^+^ and [M−117]^+^ ions shifted from 335 to 339, and from 223 to 226, respectively, demonstrating that [M−15]^+^ and [M−117]^+^ ions contained four and three carbons, respectively (Fig. 1b). Similarly, the fragment ions of [f101]^+^, [f117]^+^, [f189]^+^, [f217]^+^, [f265]^+^, and [f307]^+^ contained 2, 1, 2, 3, 1, and 3 carbons in a malic acid backbone (Fig. 1b). The cleaved TMS-carboxyl group in [M−117]^+^ could be C1 or C4 from a malic acid backbone. To determine this, position-specific ^13^C-labeled malic acid standard was prepared *via* *in vitro* sequential enzyme reactions of pyruvate kinase, phosphoenolpyruvate (PEP) carboxylase, and malate dehydrogenase supplemented with ^13^C pyruvic acid and ^13^C sodium bicarbonate (see experimental section, Fig. S1a). The analyses of TMS-derivatized [2-^13^C], [3-^13^C], and [4-^13^C]malic acids demonstrated the mass shift for [M−117]^+^ from 233 to 234 (Fig. 1d–f). However, no shift occurred in the spectrum of [1-^13^C]malic acid (Fig. 1c), indicating that the [M−117]^+^ loses the C1 in the malic acid backbone. Similarly, the following carbon positions in other fragment ions were identified: [f101]^+^ and [f189]^+^ had C2-3, [f117]^+^ had C4, [f265]^+^ had C2, and [f217]^+^ and [f307]^+^ had C2-3-4 from a malic acid backbone. The estimated structure of each fragment ions is illustrated in Fig. 2a. Although the structure of [f265]^+^ cannot be explained in a simple cleavage pattern, it is estimated that three TMS-O groups were possibly linked to the C2 of the malic acid backbone. The same approaches were applied for the survey of fragmentation in the TMS-derivatized citric, αKG, succinic, and fumaric acids (Supplementary materials 1). αKG was additionally methoxyaminated before the TMS derivatization. The fragment ions and the estimated structures of these TMS-derivatized organic acids with carbon backbone cleavage are summarized in Table 1 and Fig. 2b–e. The cleavage of TMS-carboxyl group in citric acid was successfully identified as C6 in the carbon backbone (Fig. 2b). The spectrum of αKG shows the generation of fragment ions with neither C1 nor C5 in the carbon backbone (Fig. 2c). The fragmentation of TMS-derivatized succinic and fumaric acids was estimated as Fig. 2d–e according to the structural symmetry.

(a) natural, (b) [U-^13^C], (c) [1-^13^C], (d) [2-^13^C], (e)[3-^13^C], and (f) [4-^13^C]malic acid.

(a) malic, (b) citric, (c) αKG, (d) succinic, and (e) fumaric acids. The position of demethylation in TMS group was not determined uniquely.

**Table 1:** Table 1. Fragment ions with C–C bond cleavage of TMS- and TBDMS-derivatized organic acids by EI.

Metabolites	*m*/*z*	Number of organic acid-derived carbons	Carbon skeleton	Estimated chemical formula	Estimated cleavage group
*TMS derivatization*					
Citric acid (4TMS)	273	5	C1-2-3-4-5	C_11_H_21_O_4_Si_2_	TMS-COO and TMS-OH
Citric acid (4TMS)	347	5	C1-2-3-4-5	C_13_H_27_O_5_Si_3_	TMS-COO and CH_3_
Citric acid (4TMS)	363	5	C1-2-3-4-5	C_14_H_31_O_5_Si_3_	TMS-COO
αKG (1MEOX, 2TMS)	112	4	C1-2-3-4	C_5_H_6_NO_2_	TMS-COO and TMS-O
αKG (1MEOX, 2TMS)	156	4	C2-3-4-5	C_6_H_10_NO_2_Si	TMS-COO, CH_3_ and CH_3_O
αKG (1MEOX, 2TMS)	186	4	C1-2-3-4	C_7_H_12_NO_3_Si	TMS-COO and CH_3_
Succinic acid (2TMS)	116	2	C1-2 or C3-4	C_4_H_8_O_2_Si	TMS-COO-CH_2_
Fumaric acid (2TMS)	115	2	C1-2 or C3-4	C_4_H_7_O_2_Si	TMS-COO-CH
Fumaric acid (2TMS)	143	3	C1-2-3 or C2-3-4	C_6_H_11_O_2_Si	TMS-COO
Malic acid (3TMS)	101	2	C2-3	C_4_H_9_OSi	TMS-COO, TMS-COO and CH_3_
Malic acid (3TMS)	117	1	C4	C_4_H_9_O_2_Si	Except TMS-COO
Malic acid (3TMS)	189	2	C2-3	C_7_H_17_O_2_Si_2_	TMS-COO, CH_3_ and CO
Malic acid (3TMS)	233	3	C2-3-4	C_9_H_21_O_3_Si_2_	TMS-COO
Malic acid (3TMS)	265	1	C2	C_9_H_25_O_3_Si_3_	Unknown
Malic acid (3TMS)	307	3	C2-3-4	C_11_H_27_O_4_Si_3_	CO and CH_3_
*TBDMS derivatization*					
Citric acid (4TBDMS)	431	5	C1-2-3-4-5	C_19_H_39_O_5_Si_3_	TBDMS-OH, TB and CO
Citric acid (4TBDMS)	357	5	C1-2-3-4-5	C_17_H_33_O_4_Si_2_	TBDMS-OH and TBDMS-CO
Citric acid (4TBDMS)	299	5	C1-2-3-4-5	C_13_H_23_O_4_Si_2_	TBDMS-OH, TBDMS-CO and TB
αKG (1MEOX, 2TBDMS)	156	4	C2-3-4-5	C_6_H_10_NO_2_Si	TBDMS-CO, TB and CH_3_O
αKG (1MEOX, 2TBDMS)	186	4	C1-2-3-4	C_7_H_12_NO_3_Si	TBDMS-COO and CH_3_
Malic acid (3TBDMS)	217	2	C2-3	C_9_H_21_O_2_Si_2_	TBDMS-COO, CO, TB and CH_3_
Malic acid (3TBDMS)	317	3	C2-3-4	C_15_H_33_O_3_Si_2_	TBDMS-CO
Malic acid (3TBDMS)	349	1	C2	C_15_H_37_O_3_Si_3_	Unknown
Malic acid (3TBDMS)	391	3	C2-3-4	C_17_H_39_O_4_Si_3_	TB and CO

The fragmentation of TBDMS-derivatized organic acids was also explored in GC-EI-MS (Supplementary materials 2). The spectra show the relatively abundant [M−57]^+^, which contains all carbon backbones generated by the neutral loss of *tert*-butyl (TB) group. The backbone-derived carbon atoms included in decarboxylated fragment ions were determined by analyzing the synthesized position-specific ^13^C-labeled standards in a similar way (Table 1). The decarboxylated position of TBDMS organic acids was similar to that of TMS-derivatized ones. The examples include the cleavage of C6 in citric acid, C1 or C5 of αKG, and C1 of malic acid.

### CID-induced fragmentation of TMS- and TBDMS-derivatized organic acids

**Figure 3:**
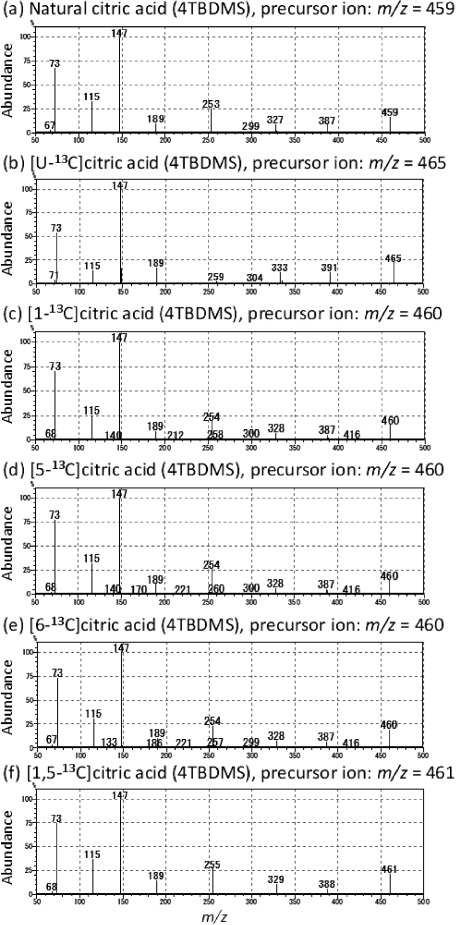
Fig. 3. CID-induced fragmentation of TBDMS-derivatized citric acid.

Additionally, CID is a promising fragmentation mode in mass spectrometry. The CID-specific fragmentation was surveyed in TMS and TBDMS-derivatized organic acids using GC-EI-MS/MS (Supplementary materials 3 and 4). Fragment ions containing all carbon backbones, *i.e.*, [M−57]^+^ and [M−15]^+^ for TBDMS- and TMS-derivatized organic acids, respectively, and relatively intense decarboxylated ions were chosen as precursor ions. The product ion scan of [f459]^+^ generated in the EI of TBDMS-derivatized citric acid, which contained all carbon backbone, produced the CID-specific ions such as [f253]^+^, [f327]^+^, and [f387]^+^ (Fig. 3a). The product ion scan of TBDMS-derivatized [U-^13^C]citric acid showed 6 mass shifts from 253 to 259 and from 327 to 333 and 4 mass shifts from 387 to 391 (Fig. 3b), indicating that [f387]^+^ contained 4 citric acid-derived carbon atoms while [f253]^+^ and [f327]^+^ contained all carbon atoms. The decarboxylated position of [f387]^+^ was investigated by the product ion scan of the various position-specific ^13^C-labeled citric acids. The *m*/*z* of product ions of TBDMS-derivatized [6-^13^C]citric acid remained at 387, demonstrating that carboxyl group of C6 was certainly lost in CID. Interestingly, the product ion scan of TBDMS-derivatized [1-^13^C] and [5-^13^C]citric acids produced ions with *m*/*z* of 387 and 388. These observations suggest that the carboxyl group of either C1 or C5 in TBDMS-citric acid was lost in CID. The assumption was validated by the presence of single ions of *m*/*z*=388 on the product ion spectra of [1,5-^13^C]citric acid. These data conclude that [f387]^+^ contained either C1-2-3-4 or C2-3-4-5. Similarly, the product ion scan of [f156]^+^ containing C1-2-3-4 of methoxyaminated and TBDMS-derivatized αKG generated in the EI produced [f112]^+^ with an additional decarboxylation in C1. Since these fragments were not observed in EI spectra, CID proved to be a complementary method to measure the ^13^C-labeling of decarboxylated fragment ions. The results are summarized in Table 2.

(a) natural, (b) [U-^13^C], (c) [1-^13^C], (d)[5-^13^C], (e)[6-^13^C] and (f)[1,5-^13^C]citric acid.

**Table 2:** Table 2. Fragment ions with C–C bond cleavage of TMS- and TBDMS-derivatized organic acids by CID.

Organic acids	Precursor ion		Product ion				
	*m*/*z*	Carbon skeleton	*m*/*z*	Number of organic acid-derived carbons	Carbon skeleton	Estimated chemical formula	Estimated cleavage group from intact molecule
*TMS derivatization*							
Citric acid (4TMS)	465	C1-2-3-4-5-6	183	5	C1-2-3-4-5	C_8_H_11_O_3_Si	TMS-COO, TMS-OH and TMS-O
Citric acid (4TMS)	465	C1-2-3-4-5-6	257	5	C1-2-3-4-5	C_10_H_17_O_4_Si_2_	CH_3_, TMS-COOH and TMS-OH
Citric acid (4TMS)	465	C1-2-3-4-5-6	347	5	C1-2-3-4-5	C_13_H_27_O_5_Si_3_	CH_3_ and TMS-COOH
Citric acid (4TMS)	465	C1-2-3-4-5-6	273	5	C1-2-3-4-5	C_11_H_21_O_4_Si_2_	TMS-OH and TMS-COOH
Citric acid (4TMS)	363	C1-2-3-4-5	183	5	C1-2-3-4-5	C_8_H_11_O_3_Si	TMS-COO, TMS-OH and TMS-O
Citric acid (4TMS)	363	C1-2-3-4-5	273	5	C1-2-3-4-5	C_11_H_21_O_4_Si_2_	TMS-COO and TMS-OH
Citric acid (4TMS)	273	C1-2-3-4-5	183	5	C1-2-3-4-5	C_8_H_11_O_3_Si	TMS-COO, TMS-OH and TMS-O
αKG (1MEOX, 2TMS)	288	C1-2-3-4-5	244	4	C1-2-3-4	C_10_H_22_O_2_NSi_2_	CH_3_O, CO, and CH_3_
αKG (1MEOX, 2TMS)	288	C1-2-3-4-5	170	4	C1-2-3-4	C_7_H_15_NO_2_Si	CH_3_O and TMS-COOH
Fumaric acid (2TMS)	245	C1-2-3-4	217	3	C1-2-3 or C2-3-4	C_8_H_17_O_3_Si_2_	CH_3_ and CO
Malic acid (3TMS)	233	C2-3-4	101	2	C2-3	C_4_H_9_OSi	TMS-COO, TMS-OOH and CH_3_
Malic acid (3TMS)	233	C2-3-4	117	1	C4	C_4_H_9_O_2_Si	Except TMS-COO
Malic acid (3TMS)	233	C2-3-4	143	3	C2-3-4	C_6_H_11_O_2_Si	TMS-COO, TMS-OH
Malic acid (3TMS)	233	C2-3-4	189	2	C2-3	C_8_H_21_OSi_2_	TMS-COO, CH_3_ and CO
Malic acid (3TMS)	335	C1-2-3-4	307	3	C2-3-4	C_11_H_27_O_4_Si_3_	CH_3_ and CO
Malic acid (3TMS)	335	C1-2-3-4	263	2	C2-3	C_10_H_27_O_2_Si_3_	CH_3_, CH_2_, CO and CO
Malic acid (3TMS)	335	C1-2-3-4	217	3	C2-3-4	C_8_H_17_O_3_Si_2_	CH_3_ and TMS-COOH
Malic acid (3TMS)	335	C1-2-3-4	117	1	C4	C_4_H_9_O_2_Si	Except TMS-COO
*TBDMS derivatization*							
Citric acid (4TBDMS)	357	C1-2-3-4-5	225	5	C1-2-3-4-5	C_11_H_17_O_3_Si	TBDMS-OH, TBDMS-COO, TBDMS-O
Citric acid (4TBDMS)	357	C1-2-3-4-5	313	4	C1-2-3-4 or C2-3-4-5	C_16_H_33_O_2_Si_2_	TBDMS-OH, TBDMS-COO, CO and CH_3_
Citric acid (4TBDMS)	431	C1-2-3-4-5	387	4	C1-2-3-4 or C2-3-4-5	C_18_H_39_O_3_Si_3_	TBDMS-OH, TB, CO, CO and CH_3_
Citric acid (4TBDMS)	459	C1-2-3-4-5-6	387	4	C1-2-3-4 or C2-3-4-5	C_18_H_39_O_3_Si_3_	TBDMS-OH, TB, CO, CO and CH_3_
αKG (1MEOX, 2TBDMS)	346	C1-2-3-4-5	186	4	C1-2-3-4	C_7_H_12_O_3_NSi	TB, and TBDMS-COO
αKG (1MEOX, 2TBDMS)	346	C1-2-3-4-5	156	4	C1-2-3-4	C_6_H_10_O_2_NSi	TB, TBDMS-COO and CH_3_O
αKG (1MEOX, 2TBDMS)	156	C1-2-3-4	112	3	C2-3-4	C_5_H_10_NSi	TB, TBDMS-COO CH_3_O, CO and CH_3_
Malic acid (3TBDMS)	419	C1-2-3-4	217	3	C2-3	C_9_H_21_O_2_Si_2_	TB, TBDMS-COO, CO and CH_3_
Malic acid (3TDBMS)	419	C1-2-3-4	391	3	C2-3-4	C_17_H_39_O_4_Si_3_	TB and CO
Malic acid (3TDBMS)	419	C1-2-3-4	403	4	C1-2-3-4	C_17_H_39_O_4_Si_3_	TB and CH_3_
Malic acid (3TDBMS)	317	C2-3-4	273	2	C2-3	C_14_H_33_OSi_2_	TBDMS-COO, CO and CH_3_

## DISCUSSION

In this study, the EI- and CID-fragmentation of TMS- and TBDMS-derivatized representative dicarboxylic and tricarboxylic acids in the Krebs cycle was explored. The analyses of the position-specific ^13^C-labeled standards prepared by *in vitro* enzymatic reactions successfully determined the position of cleaved carbon atom in the organic acid backbone (Tables 1, 2). The findings in our study are summarized as follows: (1) TMS-derivatized organic acids produced more fragment ions with C–C bond cleavage than TBDMS-derivatized ones by EI, (2) the carboxyl group next to hydroxylated carbon was primarily cleaved in EI, and (3) CID generated specific fragment ions with multiple decarboxylations.

As described in Fig. 1, various fragment ions were observed in TMS-derivatized malic acids by EI. In such case, it is possible to calculate the ^13^C-labeling of each carbon atom in organic acid backbone based on the ^13^C-labeling of several fragment ions with C–C bond cleavage.^15)^ For example, ^13^C-labeling of C1 can be calculated computationally from the ^13^C-labeling of fragment ions with C1-2-3-4 and C2-3-4 of TMS-derivatized malic acid. Even the ^13^C-labeling of each carbon atom in TMS-derivatized malic acid backbone can be determined by measuring the ^13^C-labeling of novel fragment ions identified in this study, leading to maximization of the acquirable information from a single analysis. As compared to TMS-derivatization, TBDMS-derivatized organic acids produced relatively abundant ions with all carbon backbone ([M−57]^+^) and fewer fragment ions with C–C bond cleavage. For example, fragment ions containing C4 backbone appeared on the spectra of TMS- but not TBDMS-derivatized malic acid. These data demonstrate that TMS-derivatization is beneficial for generating EI-fragment ions with C–C bond cleavage.

Although the fragmentation patterns of TMS and TBDMS-derivatized organic acids were different, a common decarboxylation rule was found in EI. The analyses of the position-specific ^13^C-labeled standards validated that C1 of malic acid and C6 of citric acid was cleaved on EI-fragment ions. This suggests that the carboxyl group linked to the hydroxylated carbons in the organic acid backbone is primarily decarboxylated. The decarboxylated fragment ions of C1 and C5 in αKG were both found in EI, implying that the carboxyl group next to methoxyaminated carbon does not have a prior decarboxylation rule.

This study also highlights the fragmentation specificity of CID in derivatized organic acids as compared to EI. As shown in Table 2, CID can cleave two TBDMS-carboxyl groups. This difference may be explained by the fact that the radical cation could be generated and stabilized even in a single decarboxylation by EI, while the ions of univalent form could be kept stable during two parts of neutral loss of carboxyl group in CID. This insight sheds light on multiple CID events by ion-trap mass spectrometry for in-depth profiling of labeling information.

In this study, the EI- and CID-fragmentation of dicarboxylic and tricarboxylic acids was surveyed. The positions of organic acid-derived carbons contained in each fragment ion were successfully identified. These findings can contribute to the development of a fundamental theory of fragmentation in derivatized organic acids as well as the improvement of ^13^C-labeling experiments for biological system.

## Notes

Mass Spectrom (Tokyo) 2019; 8(1): A0073
