# Supplementary material for: Fragmentation of Dicarboxylic and Tricarboxylic Acids in the Krebs Cycle Using GC-EI-MS and GC-EI-MS/MS
Source: Mass Spectrom (Tokyo). 2019 Aug 30;8(1):A0073. doi: 10.5702/massspectrometry.A0073 (PMC6920549; doi:10.5702/massspectrometry.A0073)

Mass Spectrometry
DOI: 10.5702/massspectrometry.A0073
Supplementary materials

Title

Fragmentation of dicarboxylic and tricarboxylic acids in the Krebs cycle using GC-EI-MS and GC-EI-MS/MS.

Authors

Nobuyuki Okahashi[1], Shuichi Kawana[2], Junko Iida[2,3], Hiroshi Shimizu[1], and Fumio Matsuda[1]

[1]Department of Bioinformatic Engineering, Graduate School of Information Science and Technology, Osaka University, 1-5 Yamadaoka, Suita, Osaka, Japan

[2]Analytical and Measuring Instruments Division, Shimadzu Corporation, 1 Nishinokyo Kuwabara-cho, Nakagyo-ku, Kyoto, Japan

[3]Osaka University Shimadzu Analytical Innovation Research Laboratory, Graduate School of Engineering, Osaka University, 2-1 Yamadaoka, Suita, Osaka, Japan

This document incudes following contents.

Supplementary figure 1 Carbon transition of enzyme reactions *in vitro*
Supplementary materials 1 MS spectra of EI-fragments of TMS derivatives
Supplementary materials 2 MS spectra of EI-fragments of TBDMS derivatives
Supplementary materials 3 MS/MS spectra of CID-fragments of TMS derivatives
Supplementary materials 4 MS/MS spectra of CID-fragments of TBDMS derivatives

Supplementary figure 1

Carbon transition of enzyme reactions *in vitro* of (a) malic and (b) citric acid.

(a)

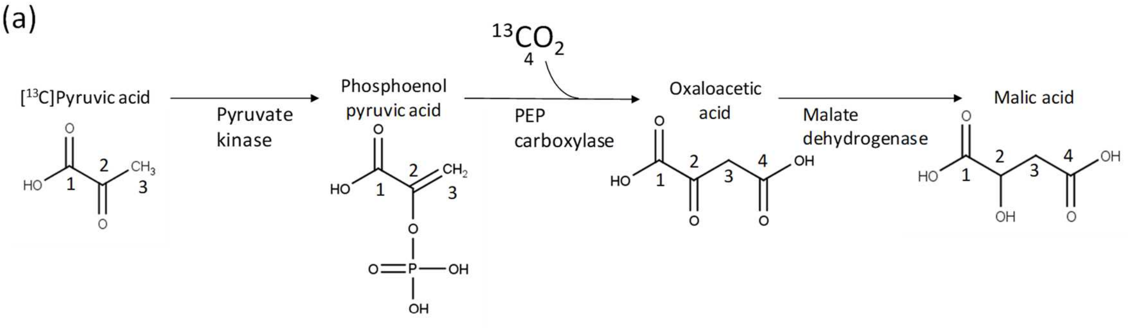

(b)

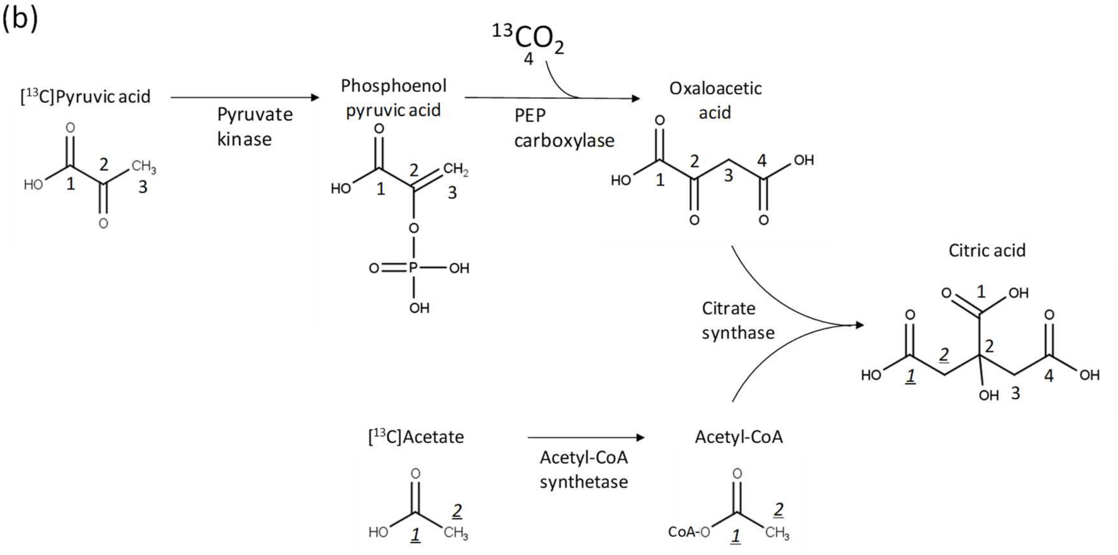

Supplementary materials 1 MS spectra of EI-fragments of TMS derivatives

Natural citric acid (4TMS)

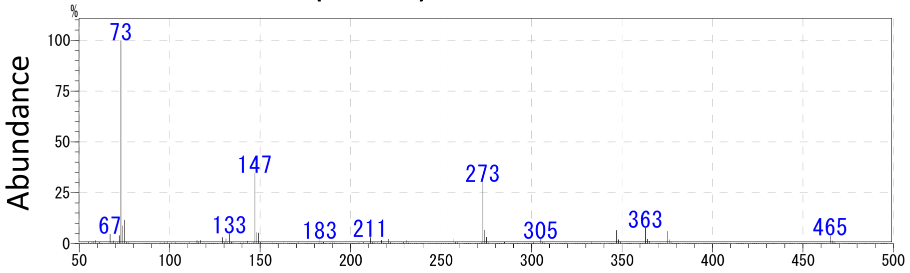

[1-^{13}C]citric acid (4TMS)

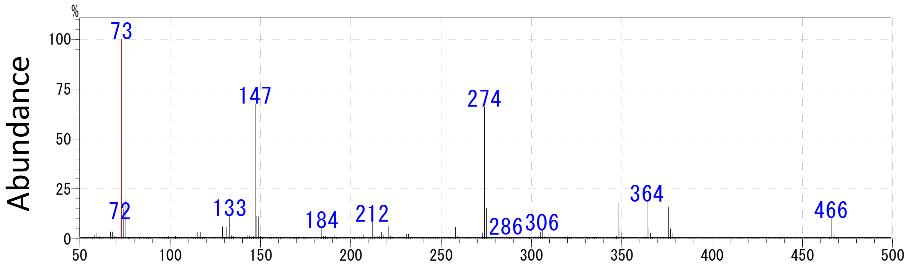

[5-^{13}C]citric acid (4TMS)

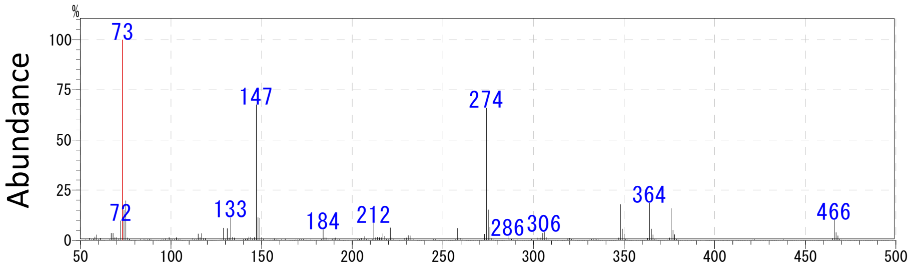

[6-^{13}C]citric acid (4TMS)

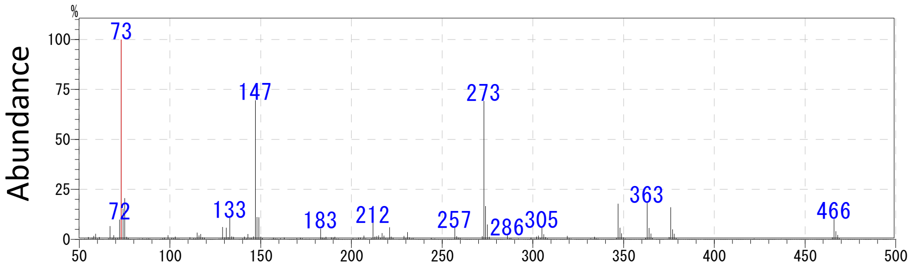

[U-^{13}C]citric acid (4TMS)

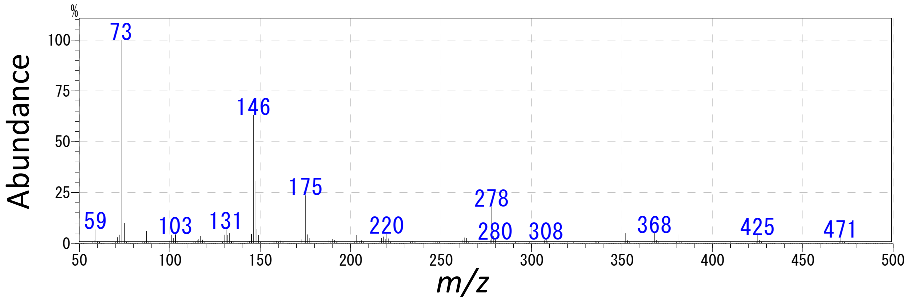

Natural α-ketoglutaric acid (1MEOX and 2TMS)

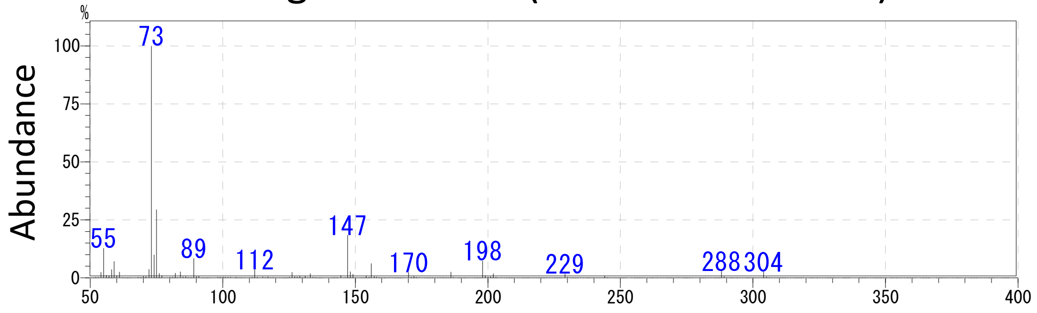

[1,2,3,4-^{13}C]α-ketoglutaric acid (1MEOX and 2TMS)

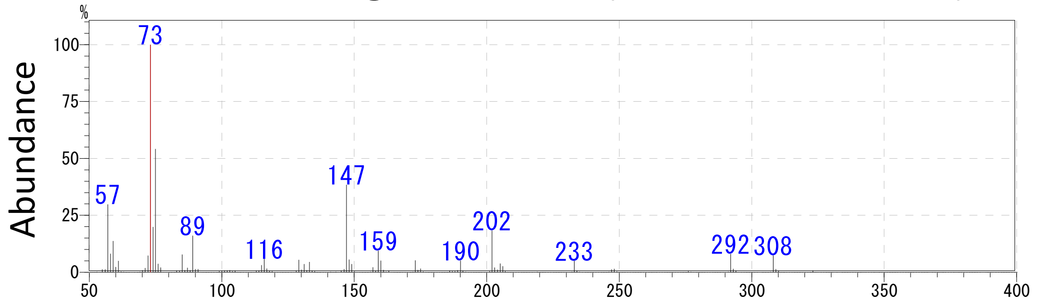

[U-^{13}C]α-ketoglutaric acid (1MEOX and 2TMS)

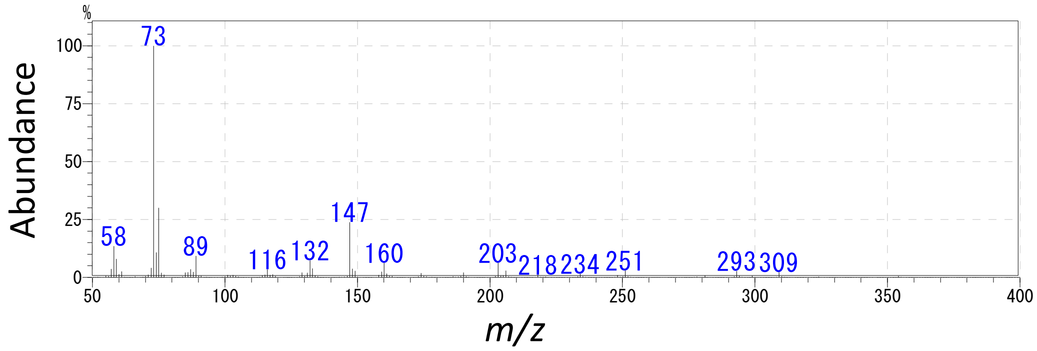

Natural succinic acid (2TMS)

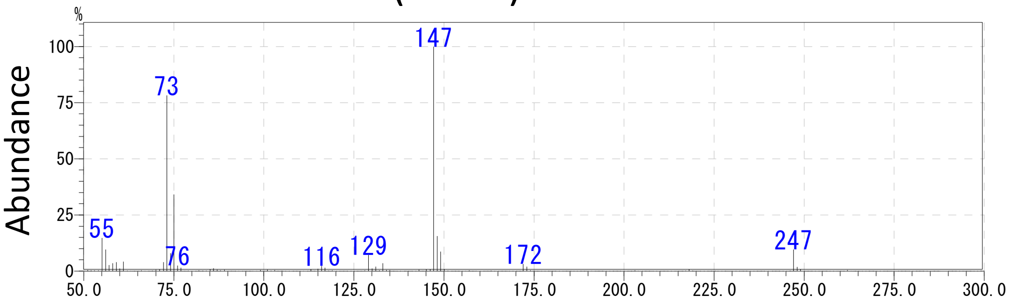

[U-^{13}C]succinic acid (2TMS)

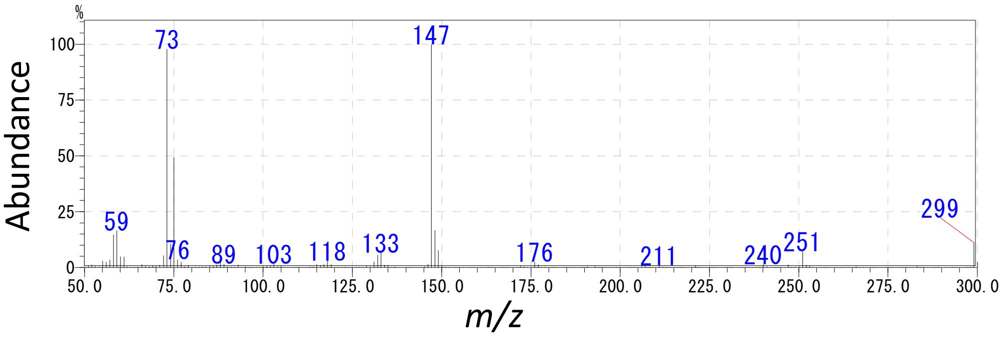

Natural fumaric acid (2TMS)

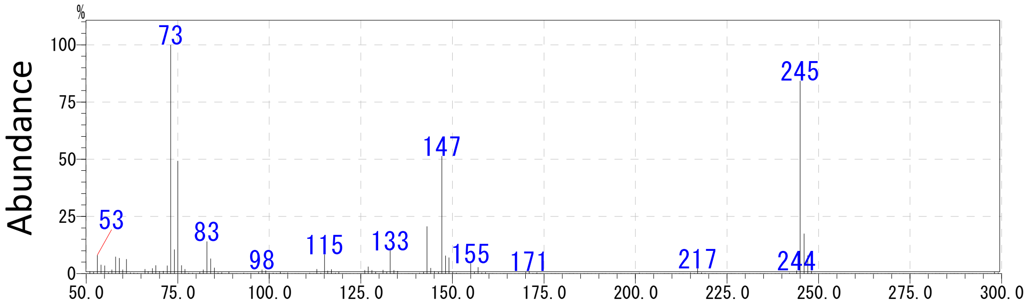

[U-^{13}C]fumaric acid (2TMS)

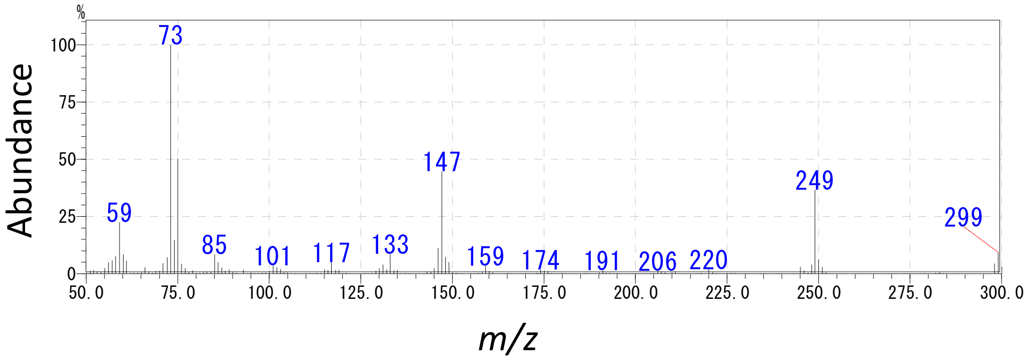

Natural malic acid (3TMS)

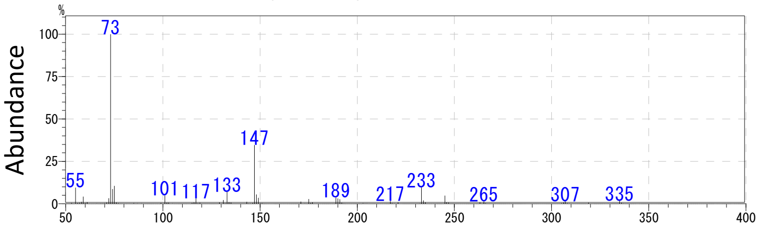

[1-^{13}C]malic acid (3TMS)

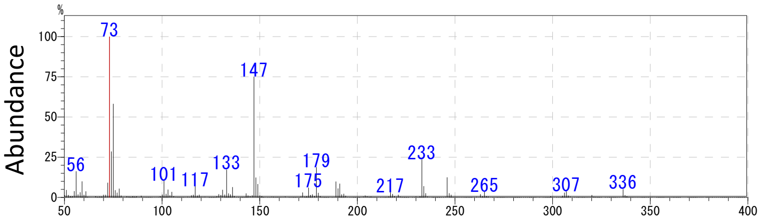

[2-^{13}C]malic acid (3TMS)

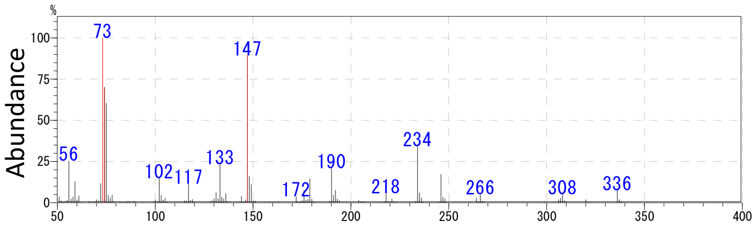

[3-^{13}C]malic acid (3TMS)

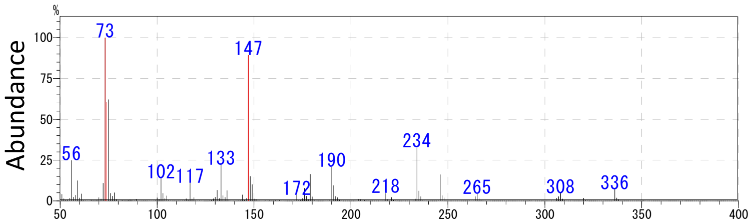

[4-^{13}C]malic acid (3TMS)

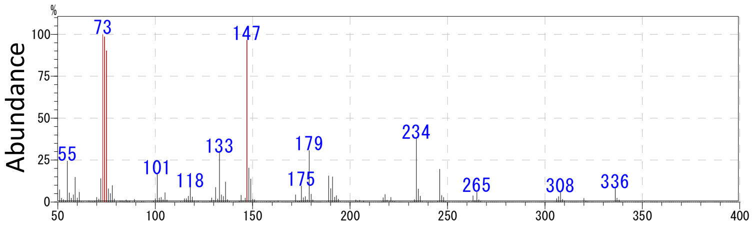

[U-^{13}C]malic acid (3TMS)

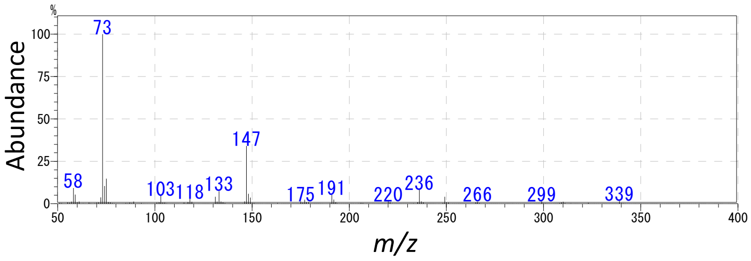

Supplementary materials 2 MS spectra of EI-fragments of TBDMS derivatives

Natural citrate (4TBDMS)

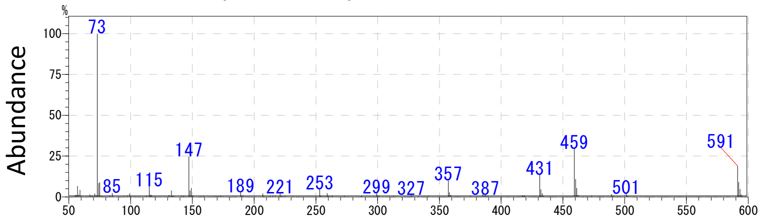

[1-^{13}C]citric acid (4TBDMS)

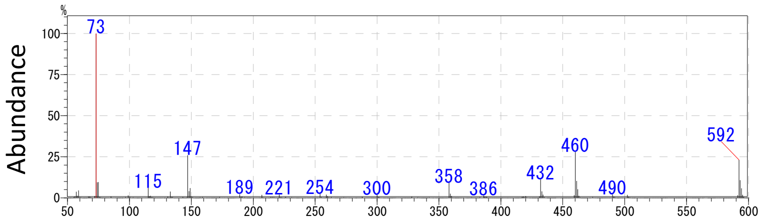

[5-^{13}C]citric acid (4TBDMS)

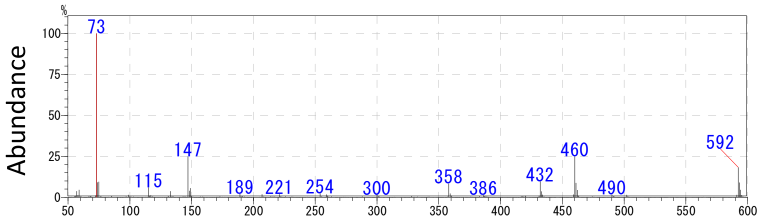

[6-^{13}C]citric acid (4TBDMS)

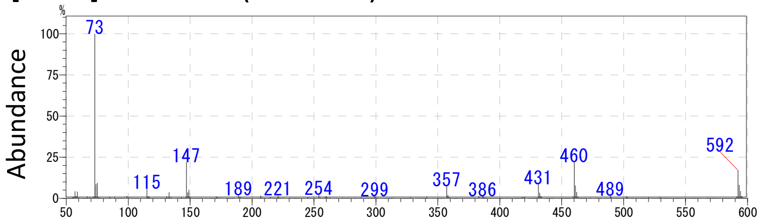

[1,5-^{13}C]citric acid (4TBDMS)

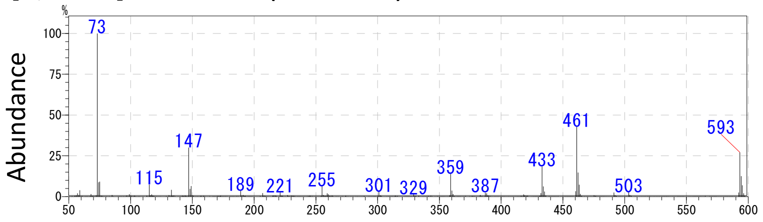

[U-^{13}C]citric acid (4TBDMS)

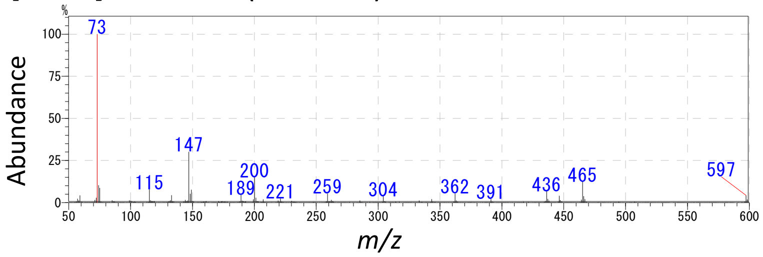

Natural α-ketoglutaric acid (1MEOX and 2TBDMS)

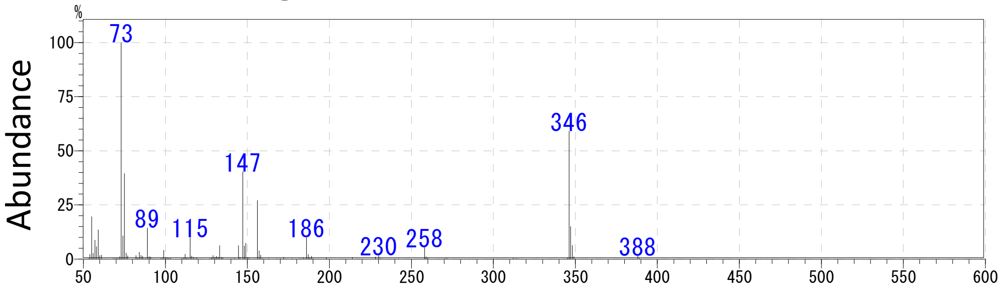

[1,2,3,4-^{13}C]α-ketoglutaric acid (1MEOX and 2TBDMS)

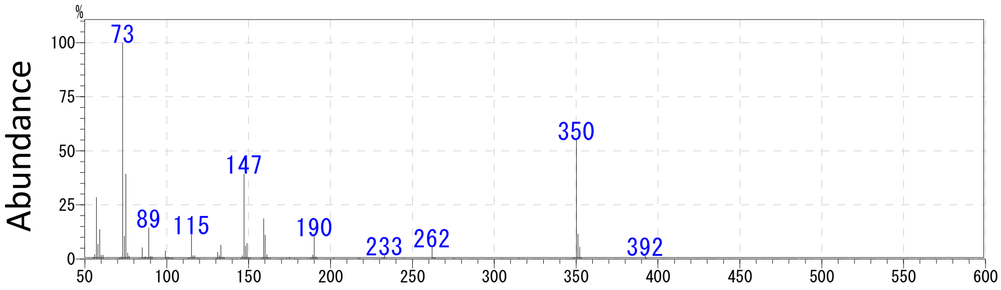

[U-^{13}C]α-ketoglutaric acid (1MEOX and 2TBDMS)

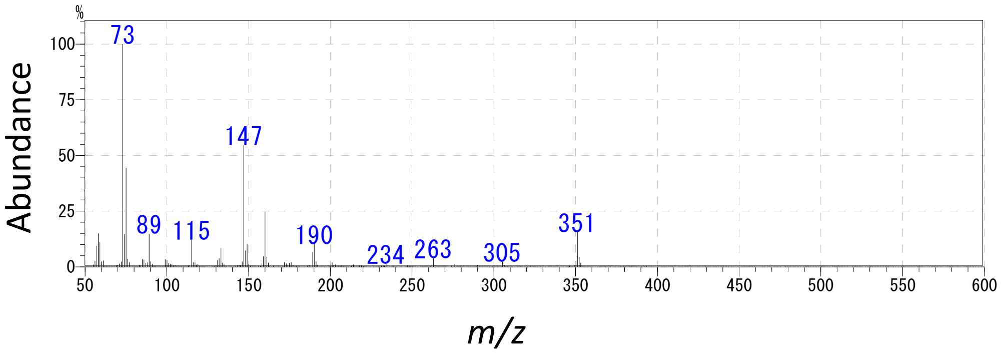

Natural succinic acid (2TBDMS)

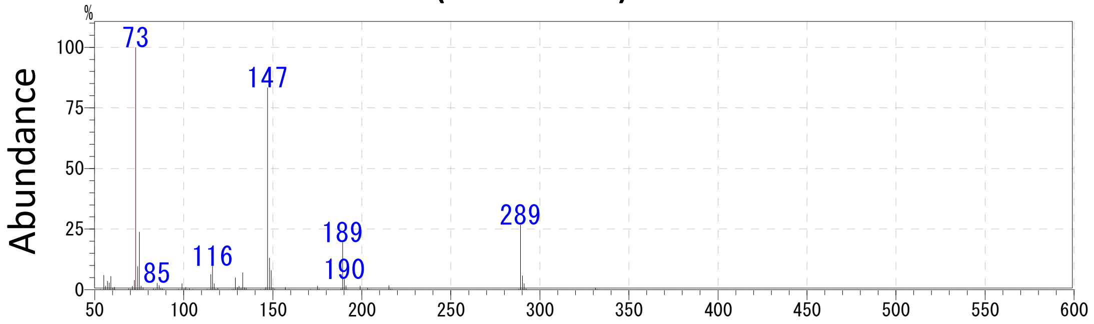

[U-^{13}C]succinic acid (2TBDMS)

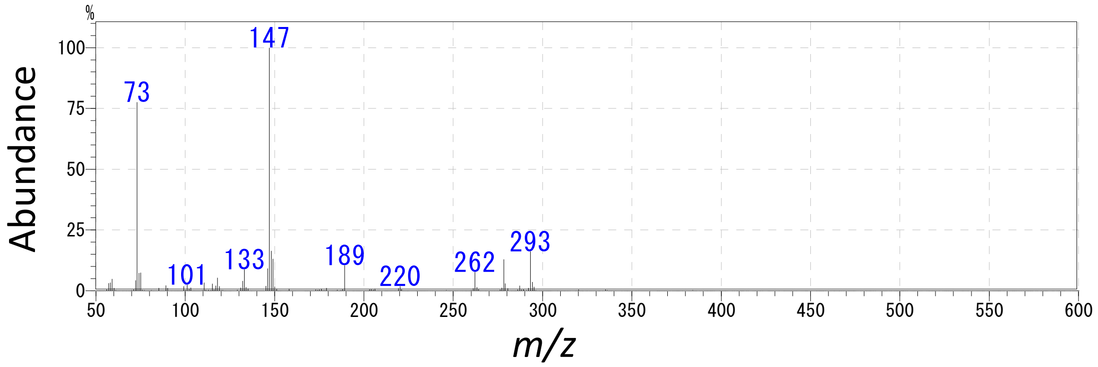

Natural fumaric acid (2TBDMS)

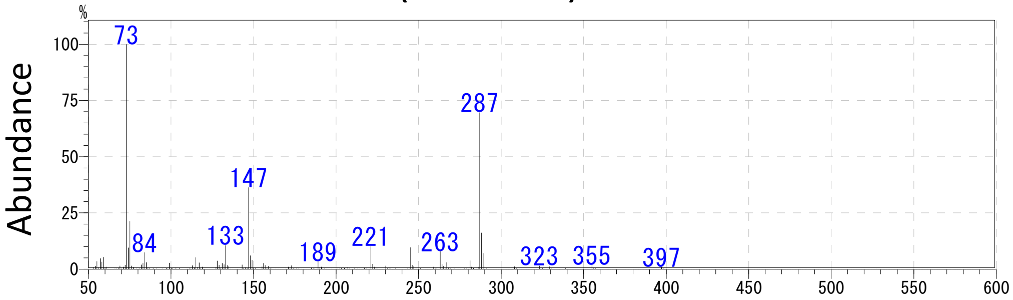

[U-^{13}C]fumaric acid (2TBDMS)

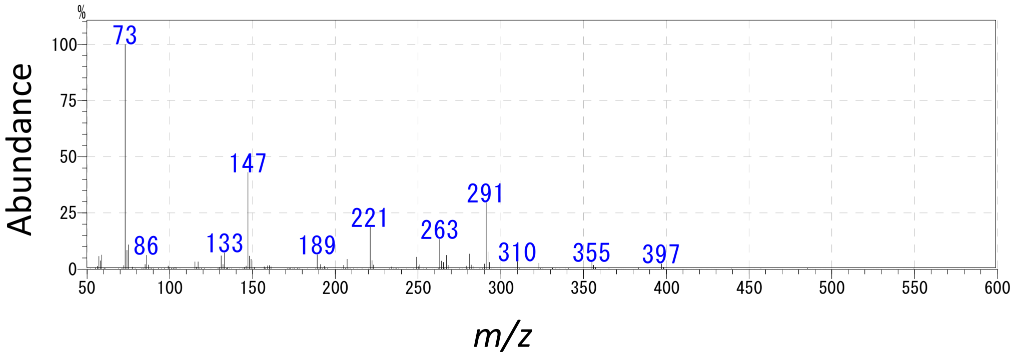

Natural malic acid (3TBDMS)

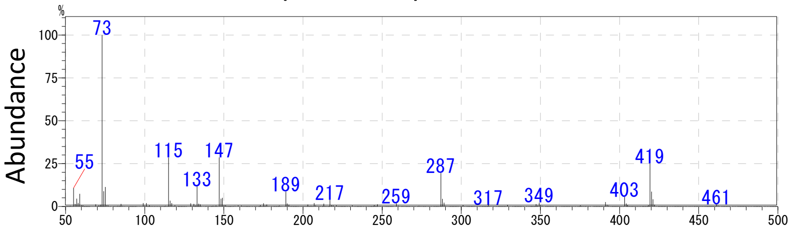

[1-^{13}C]malic acid (3TBDMS)

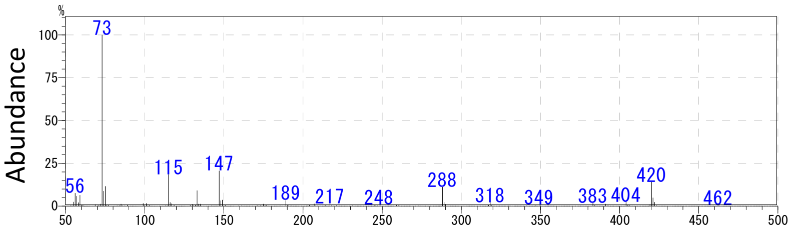

[2-^{13}C]malic acid (3TBDMS)

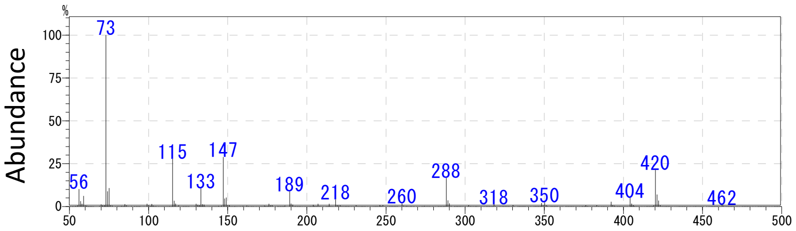

[3-^{13}C]malic acid (3TBDMS)

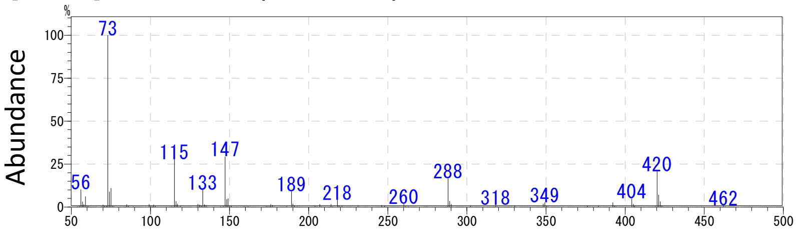

[4-^{13}C]malic acid (3TBDMS)

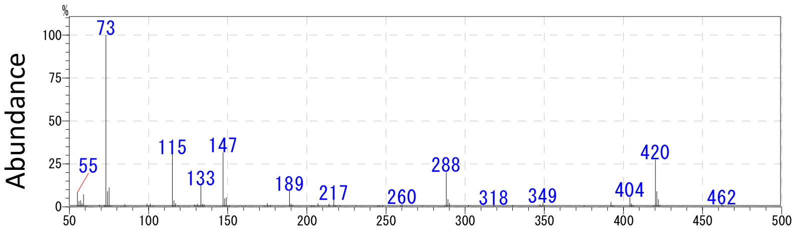

[U-^{13}C]malic acid (3TBDMS)

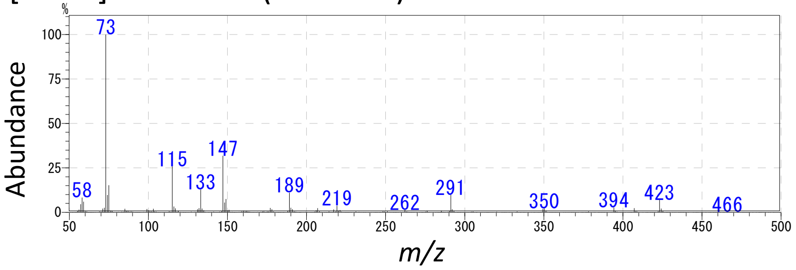

Supplementary materials 3 MS/MS spectra of CID-fragments of TMS derivatives

Natural Citric acid (4TMS), precursor ion: m/z = 465

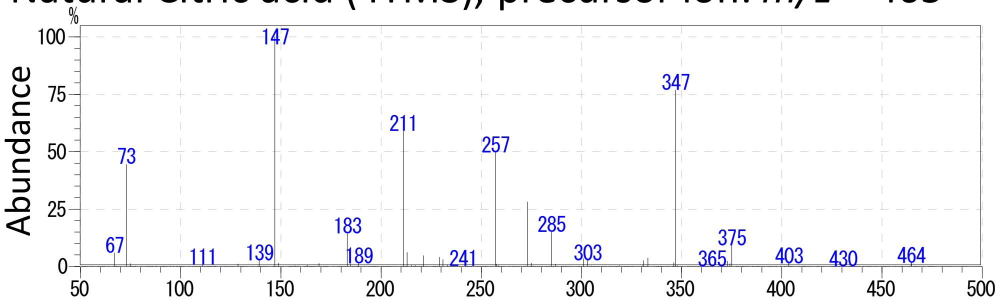

[U-^{13}C]citric acid (4TMS), precursor ion: m/z = 471

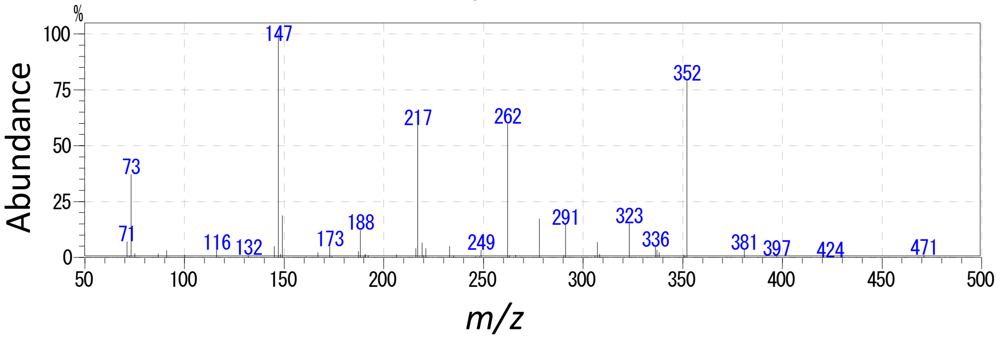

Natural Citric acid (4TMS), precursor ion: m/z = 363

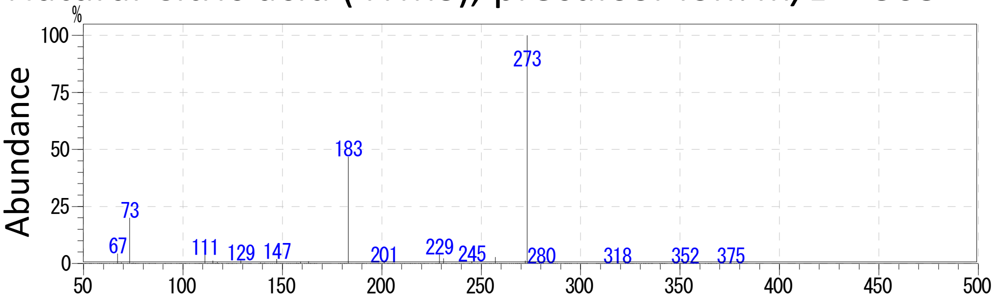

[U-^{13}C]citric acid (4TMS), precursor ion: m/z = 368

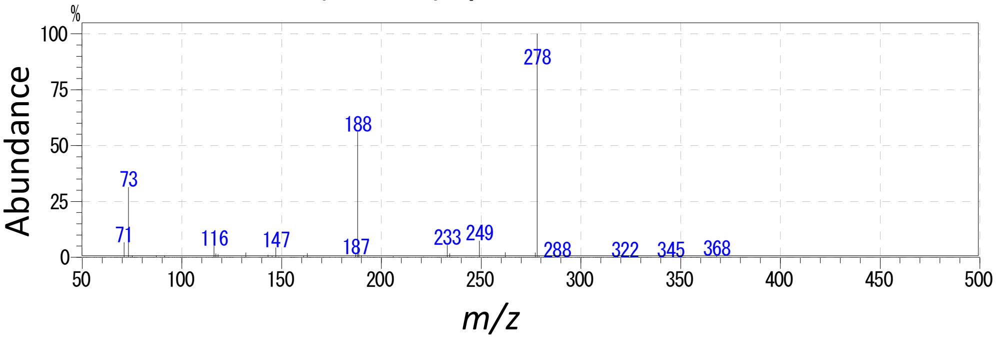

Natural Citric acid (4TMS), precursor ion: *m/z* = 273

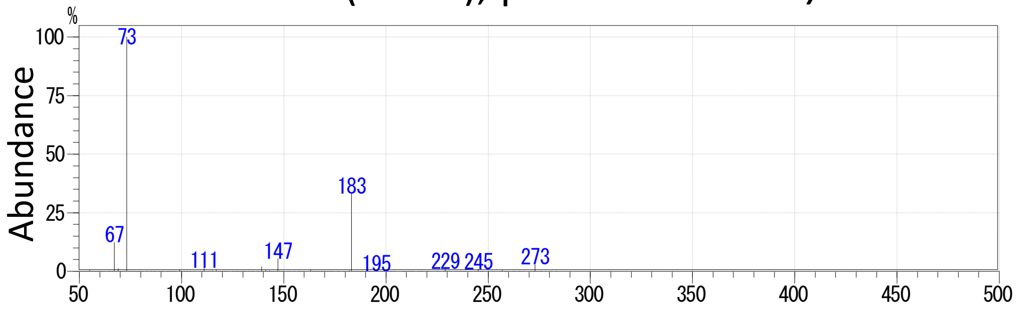

[U-^{13}C]citric acid (4TMS), precursor ion: *m/z* = 278

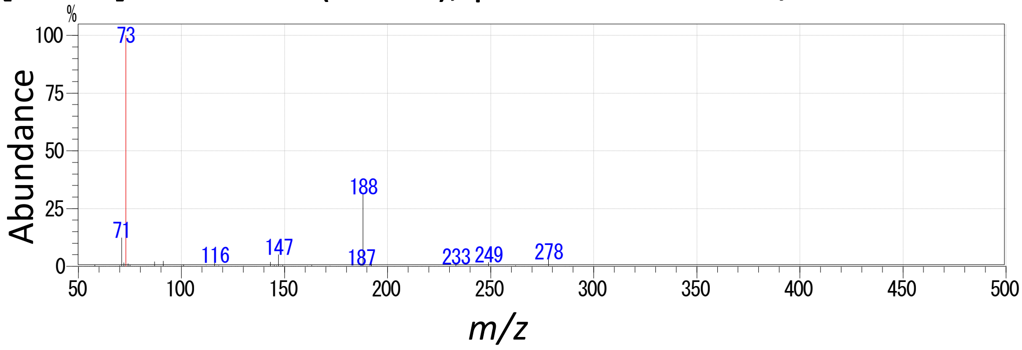

Natural α-ketoglutaric acid (1MEOX and 2TMS), precursor ion: *m/z* = 288

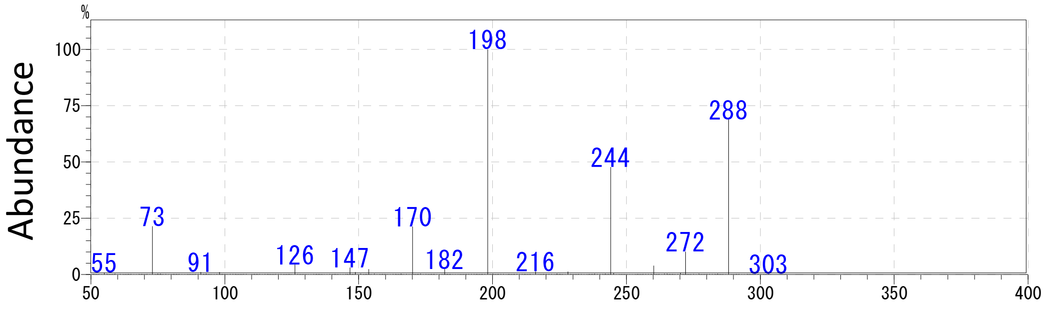

[1,2,3,4-^{13}C]α-ketoglutaric acid (1MEOX and 2TMS), precursor ion: *m/z* = 292

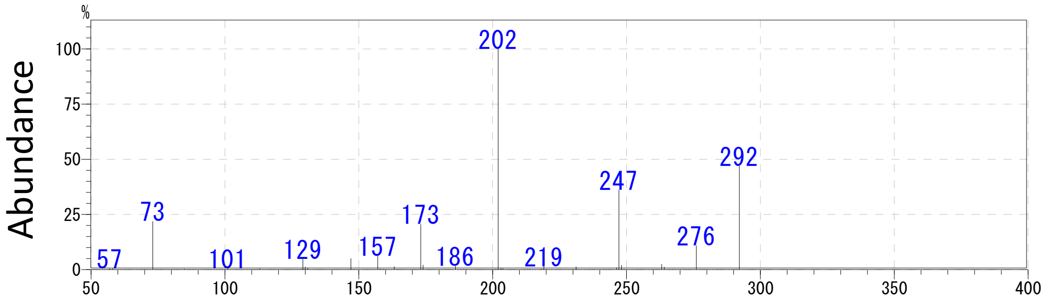

[U-^{13}C]α-ketoglutaric acid (1MEOX and 2TMS), precursor ion: *m/z* = 293

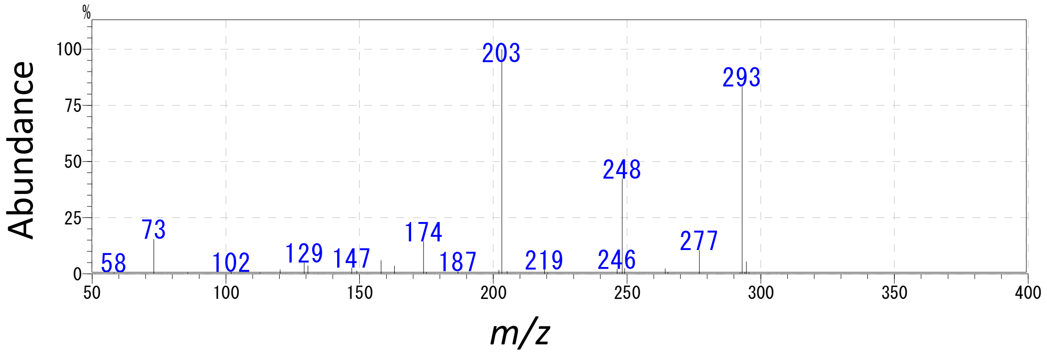

Natural succinic acid (2TMS), precursor ion: *m/z* = 247

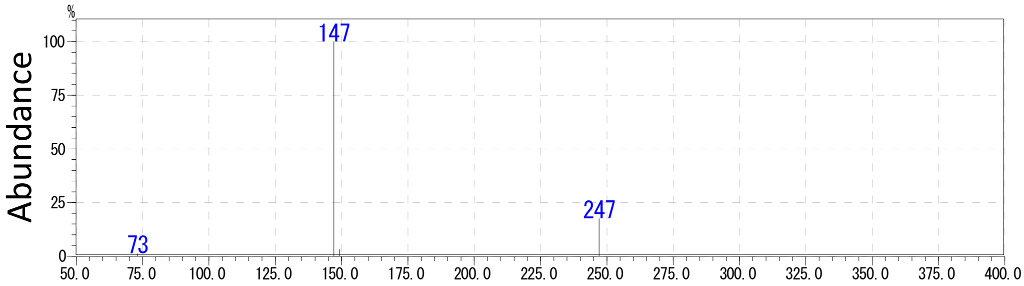

[U-^{13}C]succinic acid (4TMS), precursor ion: *m/z* = 251

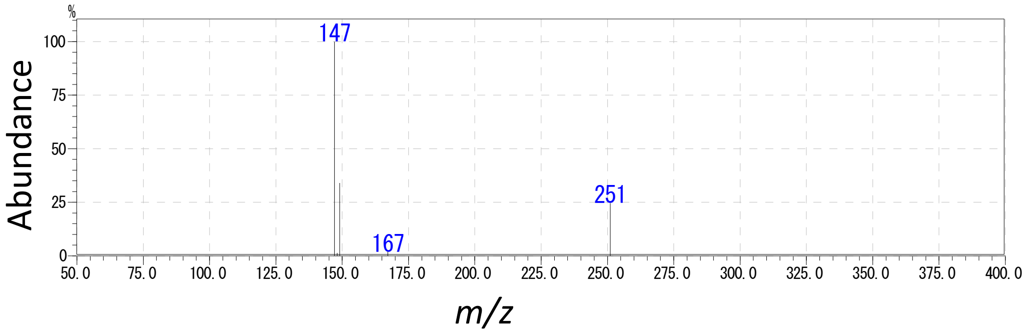

Natural fumaric acid (2TMS), precursor ion: m/z = 245

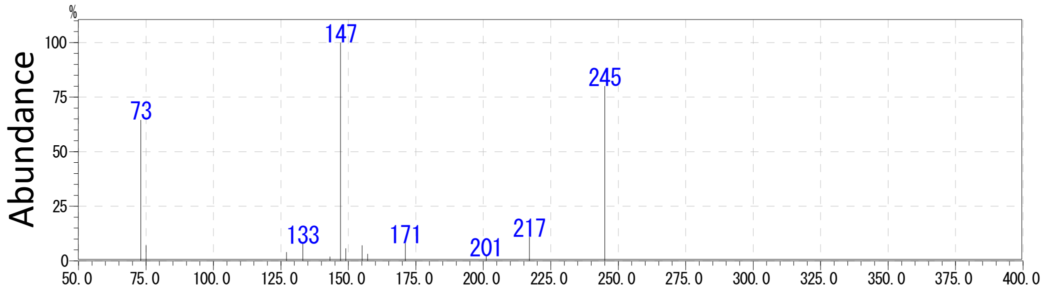

[U-^{13}C]fumaric acid (4TMS), precursor ion: m/z = 249

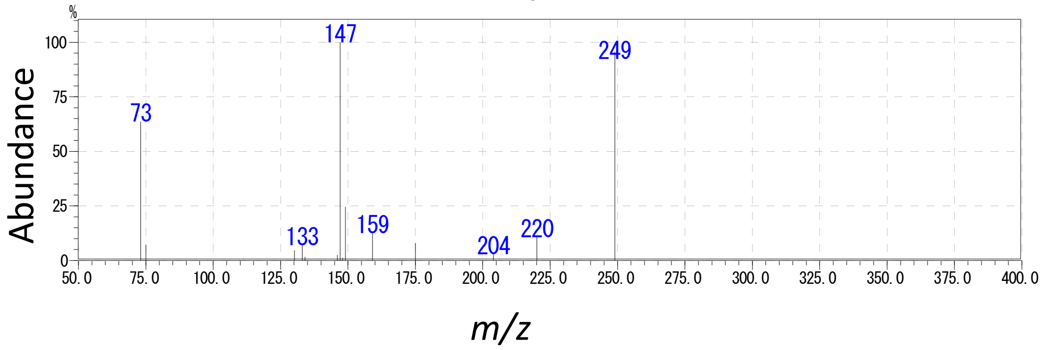

Natural malic acid (3TMS), precursor ion: *m/z* = 335

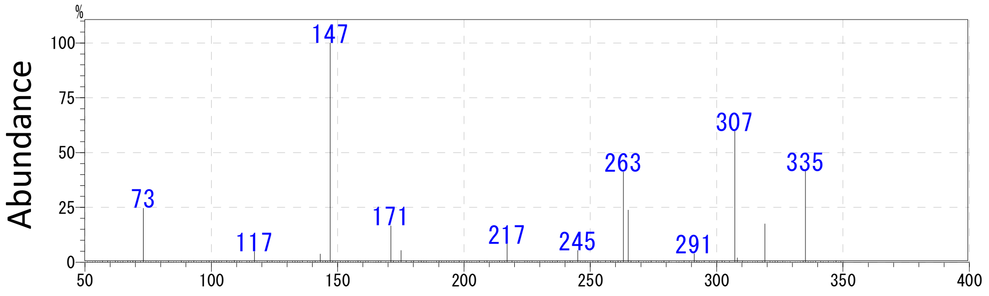

[2-^{13}C]malic acid (3TMS), precursor ion: *m/z* = 336

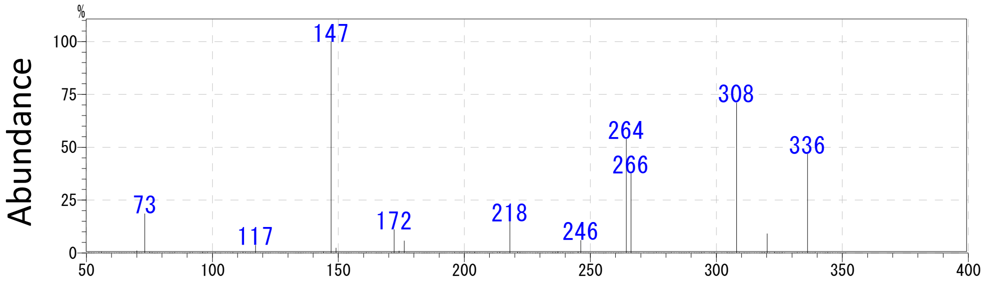

[3-^{13}C]malic acid (3TMS), precursor ion: *m/z* = 336

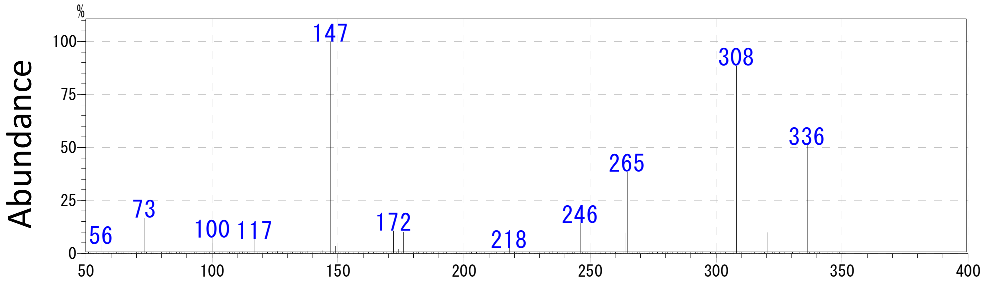

[4-^{13}C]malic acid (3TMS), precursor ion: *m/z* = 336

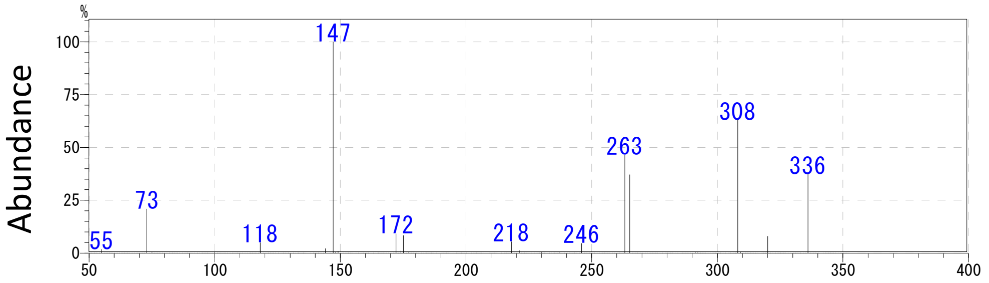

[U-^{13}C]malic acid (3TMS), precursor ion: *m/z* = 339

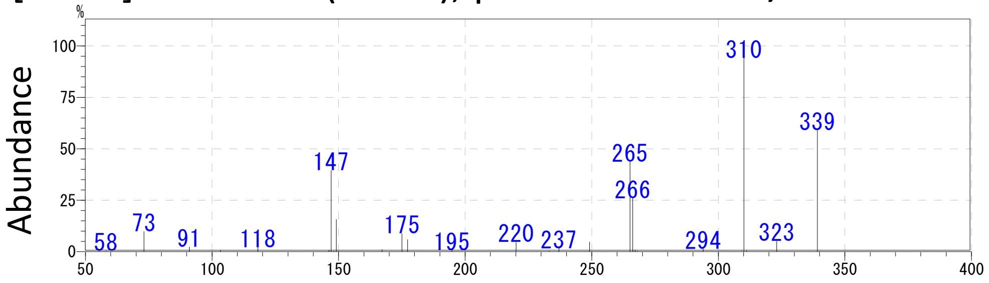

m/z

Natural malic acid (3TMS), precursor ion: *m/z* = 233

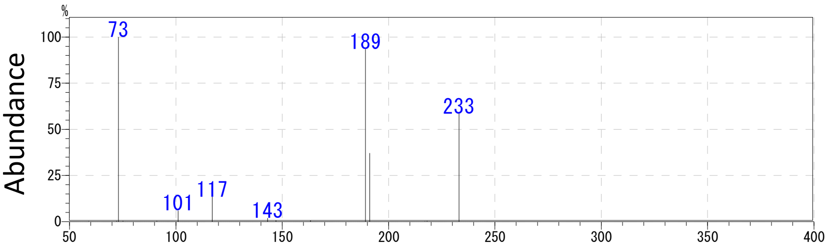

[1-^{13}C]malic acid (3TMS), precursor ion: *m/z* = 233

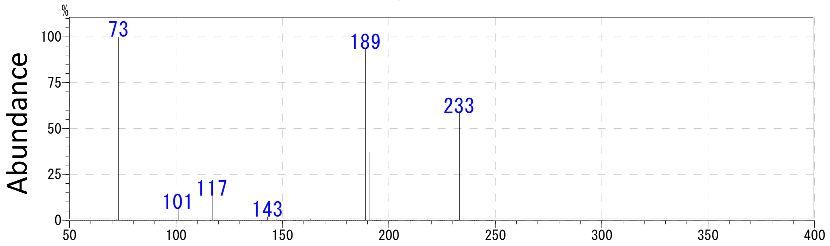

[2-^{13}C]malic acid (3TMS), precursor ion: *m/z* = 234

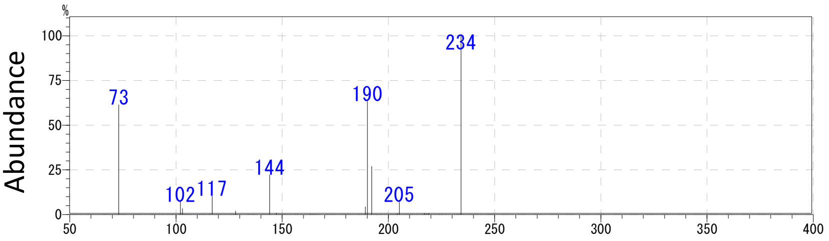

[3-^{13}C]malic acid (3TMS), precursor ion: *m/z* = 234

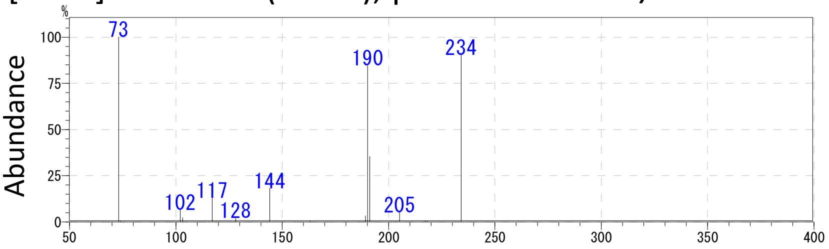

[4-^{13}C]malic acid (3TMS), precursor ion: *m/z* = 234

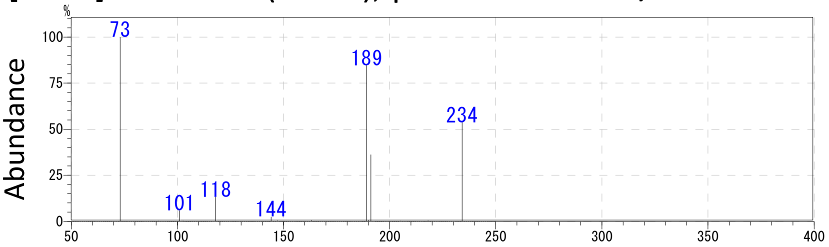

[U-^{13}C]malic acid (3TMS), precursor ion: *m/z* = 236

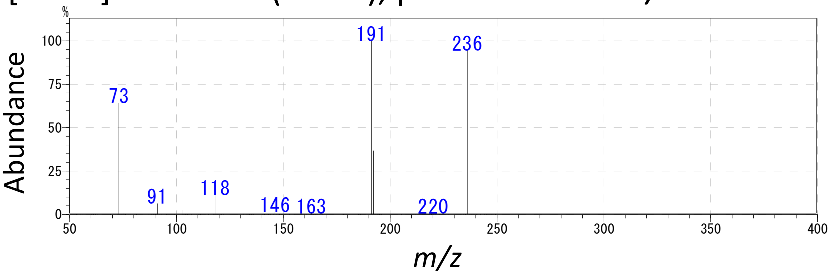

Supplementary materials 4 MS/MS spectra of CID-fragments of TBDMS derivatives

Natural citric acid (4TBDMS), precursor ion: *m/z* = 459

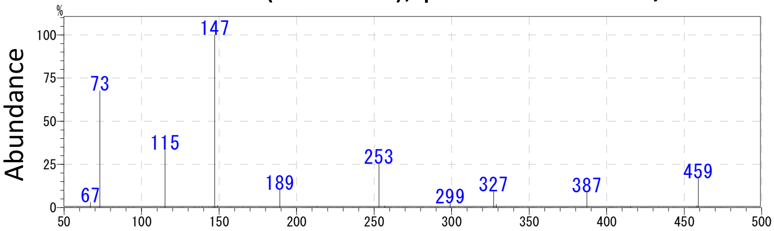

[1-^{13}C]citric acid (4TBDMS), precursor ion: *m/z* = 460

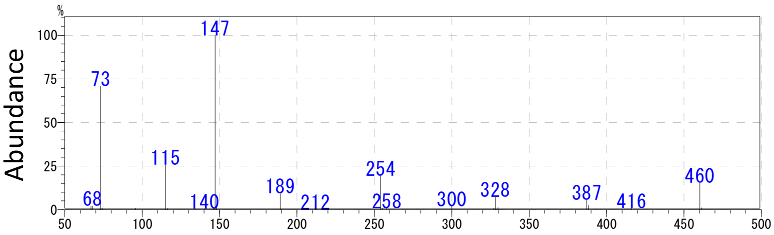

[5-^{13}C]citric acid (4TBDMS), precursor ion: *m/z* = 460

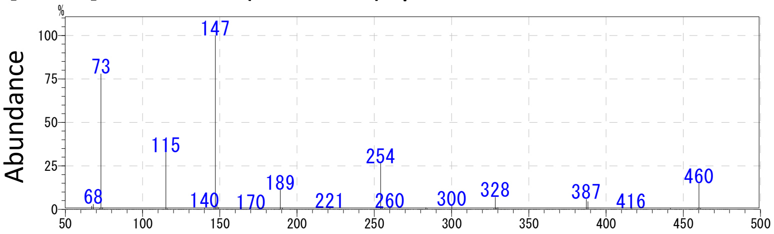

[6-^{13}C]citric acid (4TBDMS), precursor ion: *m/z* = 460

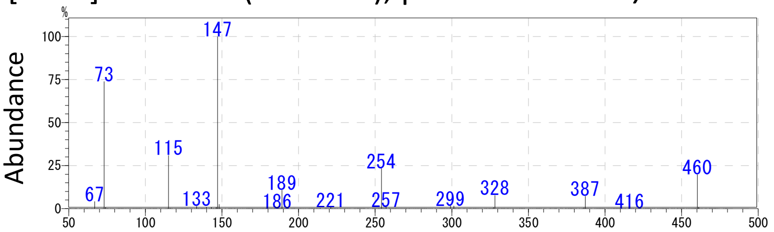

[1,5-^{13}C]citric acid (4TBDMS), precursor ion: *m/z* = 461

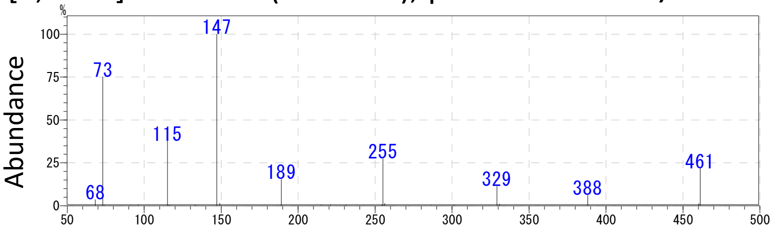

[U-^{13}C]citric acid (4TBDMS), precursor ion: *m/z* = 465

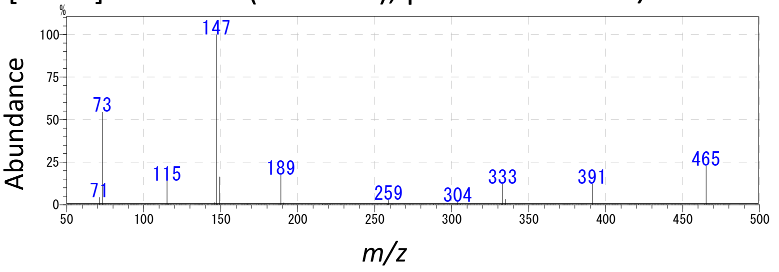

Natural citric acid (4TBDMS), precursor ion: *m/z* = 357

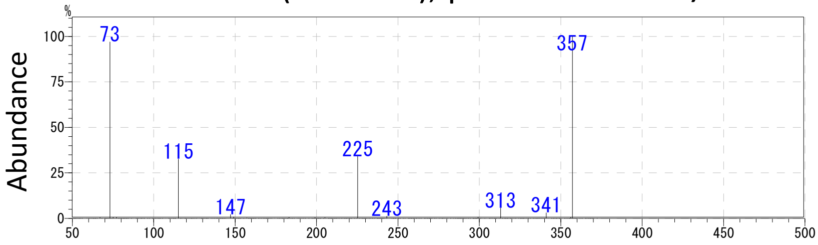

[1-^{13}C]citric acid (4TBDMS), precursor ion: *m/z* = 358

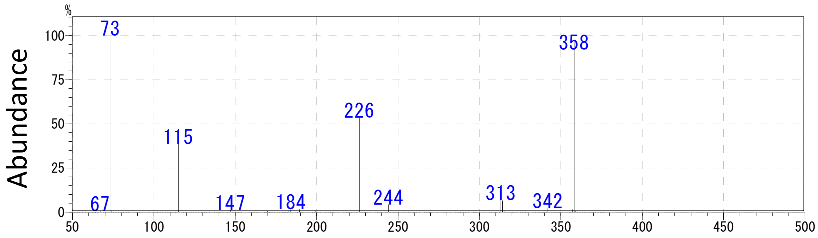

[5-^{13}C]citric acid (4TBDMS), precursor ion: *m/z* = 358

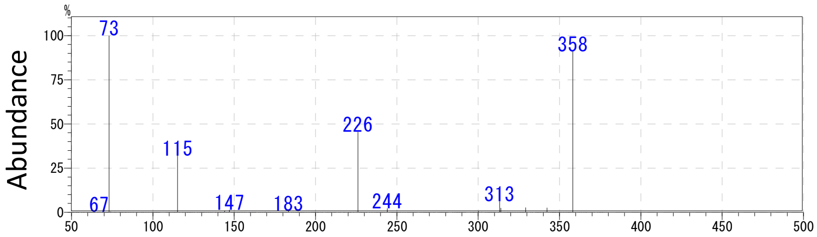

[6-^{13}C]citric acid (4TBDMS), precursor ion: *m/z* = 357

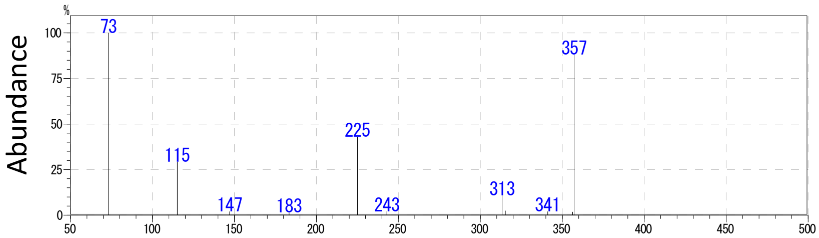

[1,5-^{13}C]citric acid (4TBDMS), precursor ion: *m/z* = 359

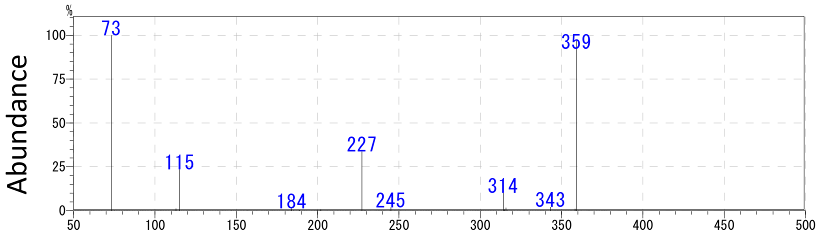

[U-^{13}C]citric acid (4TBDMS), precursor ion: *m/z* = 362

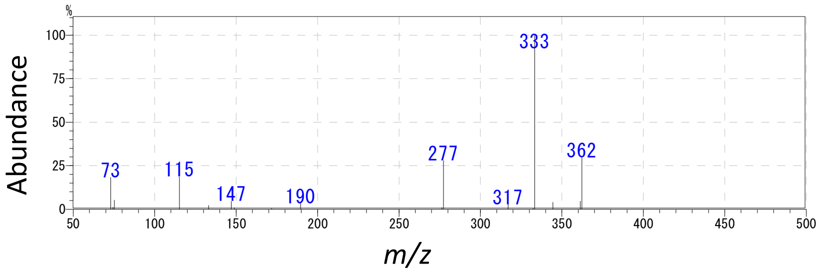

Natural α-ketoglutaric acid (1MEOX and +2TBDMS), precursor ion: *m/z* = 346

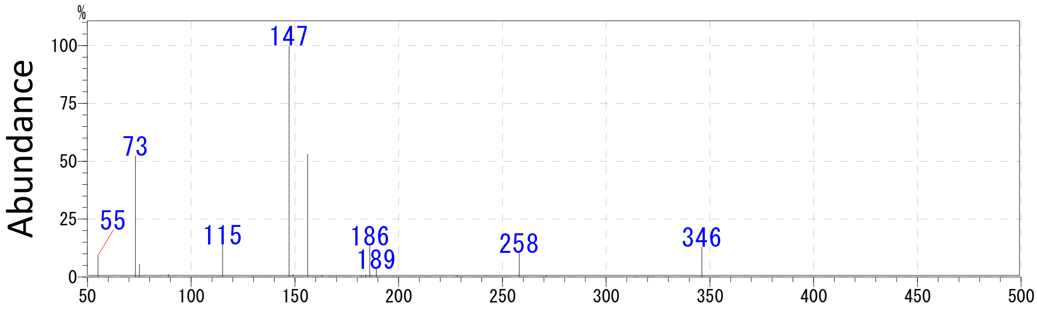

[1,2,3,4-^{13}C]α-ketoglutaric acid (1MEOX and 2TBDMS), precursor ion: *m/z* = 350

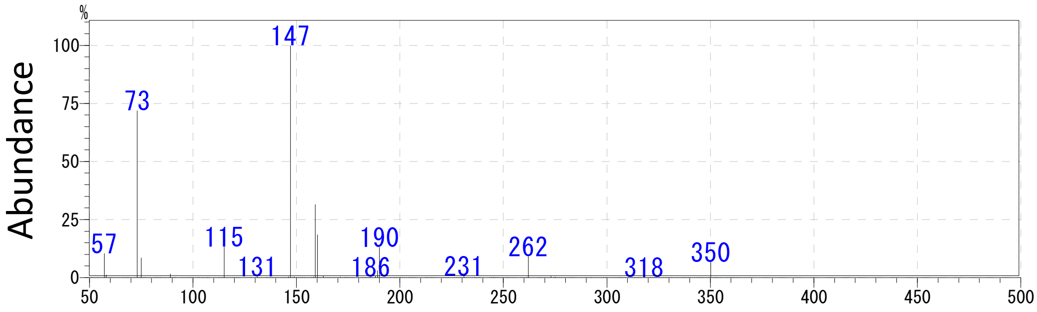

[U-^{13}C]α-ketoglutaric acid (1MEOX and 2TBDMS), precursor ion: *m/z* = 351

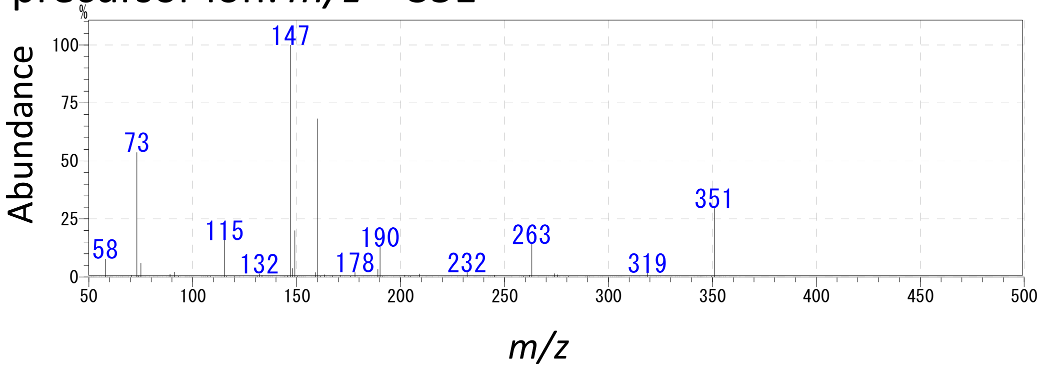

Natural α-ketoglutaric acid (1MEOX and 2TBDMS), precursor ion: *m/z* = 156

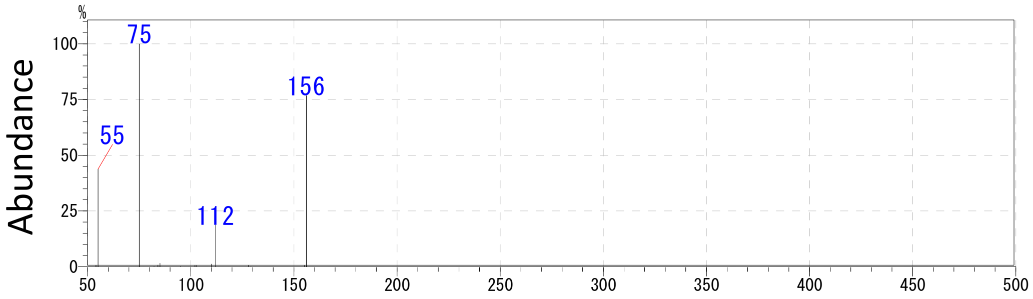

[1,2,3,4-^{13}C]α-ketoglutaric acid (1MEOX and 2TBDMS), precursor ion: *m/z* = 160

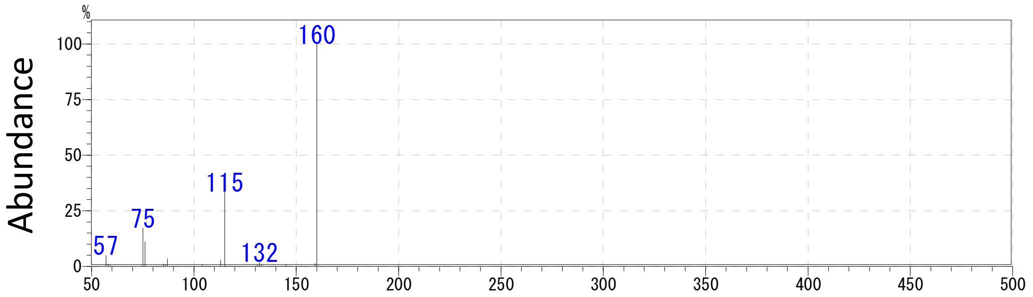

[U-^{13}C]α-ketoglutaric acid (1MEOX and 2TBDMS), precursor ion: *m/z* = 160

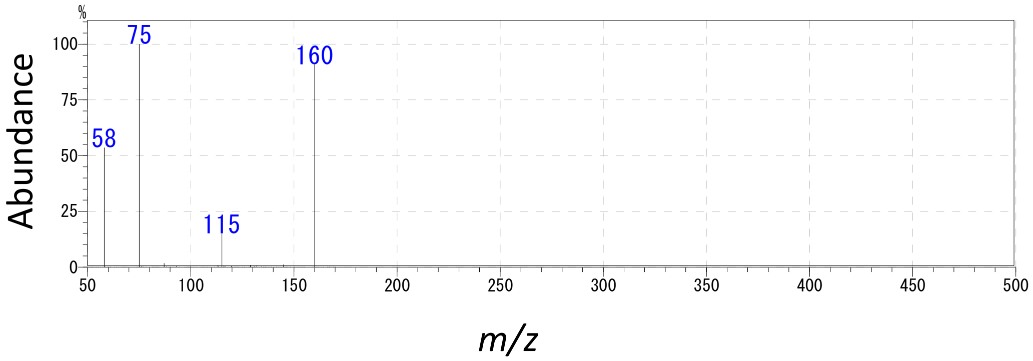

Natural succinic acid (2TBDMS), precursor ion: *m/z* = 289

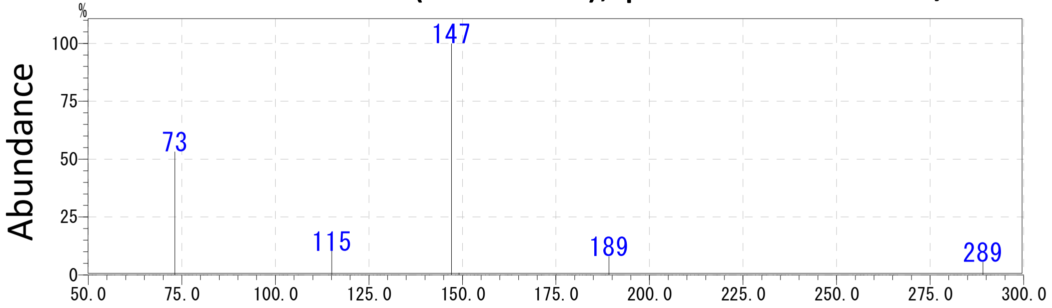

[U-^{13}C]succinic acid (2TBDMS), precursor ion: *m/z* = 293

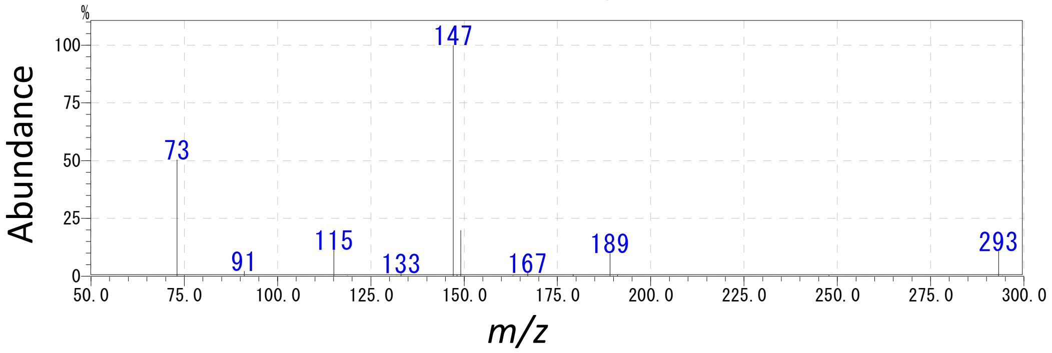

Natural fumaric acid (2TBDMS), precursor ion: *m/z* = 287

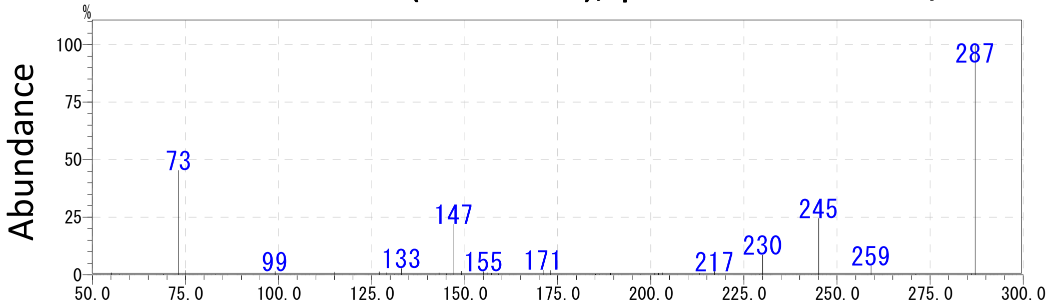

[U-^{13}C]fumaric acid (2TBDMS), precursor ion: *m/z* = 291

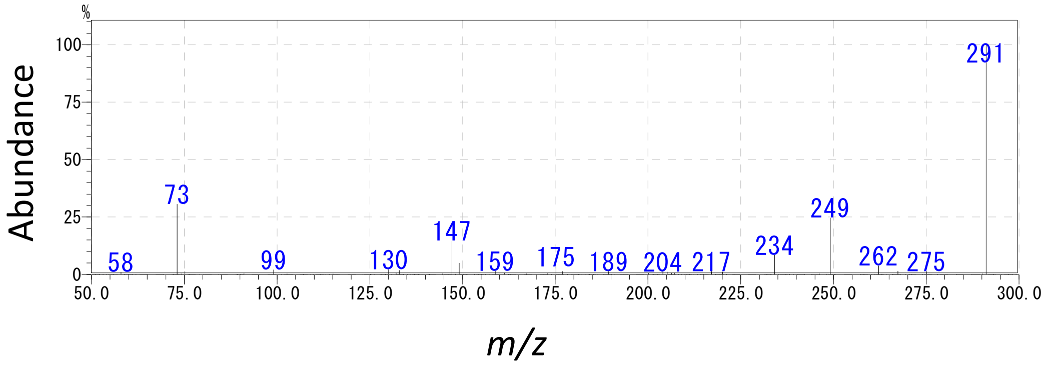

Natural malic acid (3TBDMS), precursor ion: *m/z* = 419

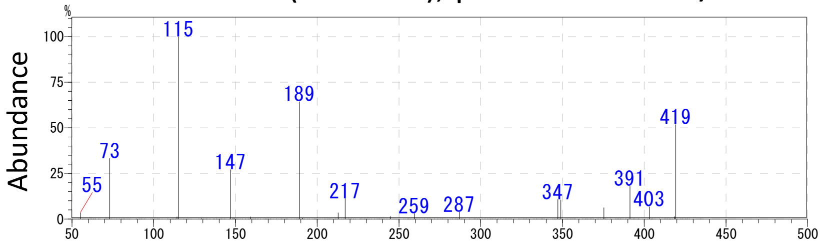

[1-^{13}C]malic acid (3TBDMS), precursor ion: *m/z* = 420

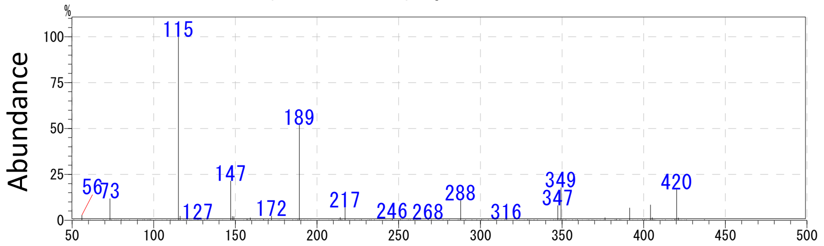

[2-^{13}C]malic acid (3TBDMS), precursor ion: *m/z* = 420

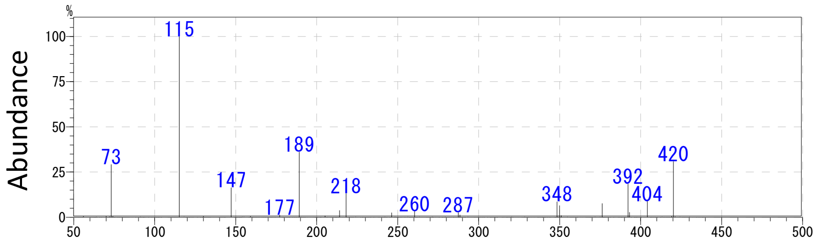

[3-^{13}C]malic acid (3TBDMS), precursor ion: *m/z* = 420

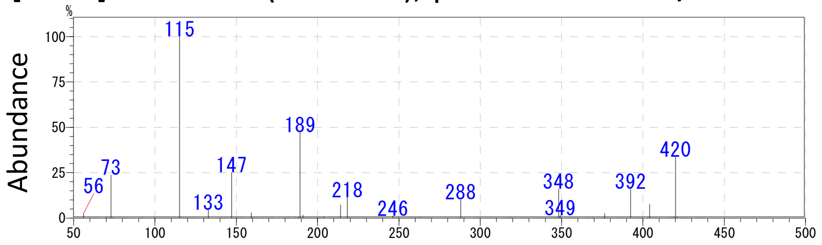

[4-^{13}C]malic acid (3TBDMS), precursor ion: *m/z* = 420

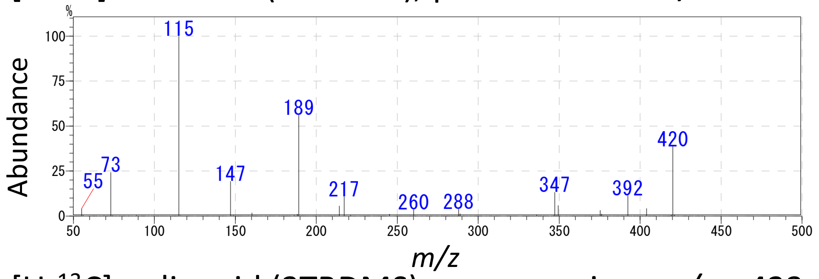

[U-^{13}C]malic acid (3TBDMS), precursor ion: *m/z* = 423

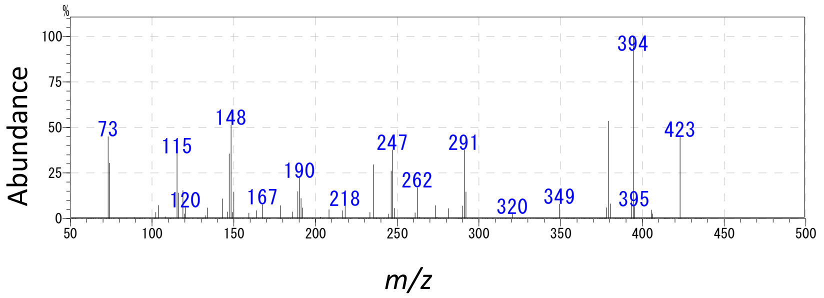

Natural malic acid (3TBDMS), precursor ion: *m/z* = 317

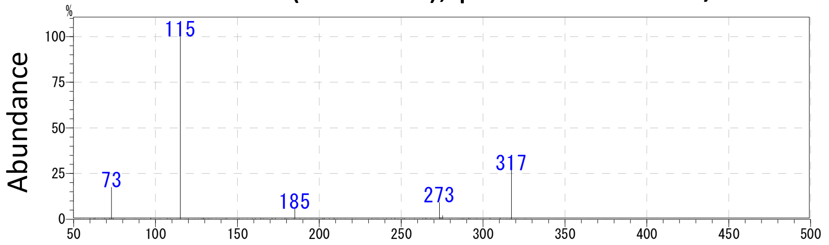

[1-^{13}C]malic acid (3TBDMS), precursor ion: *m/z* = 317

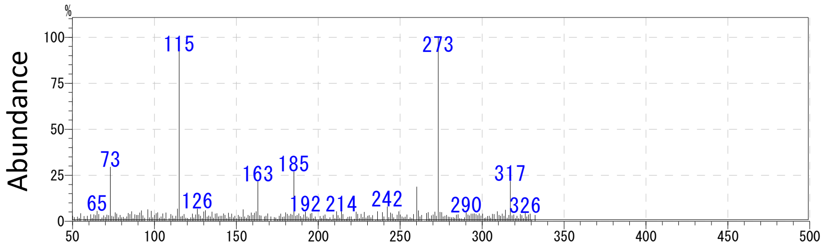

[2-^{13}C]malic acid (3TBDMS), precursor ion: *m/z* = 318

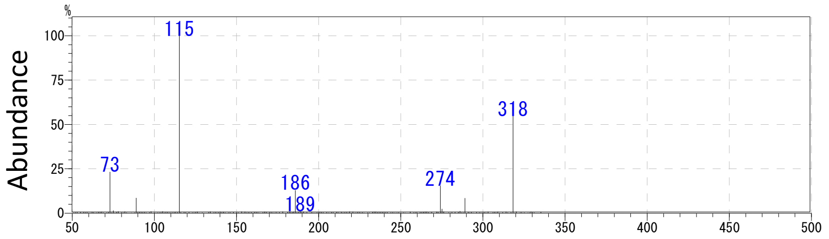

[3-^{13}C]malic acid (3TBDMS), precursor ion: *m/z* = 318

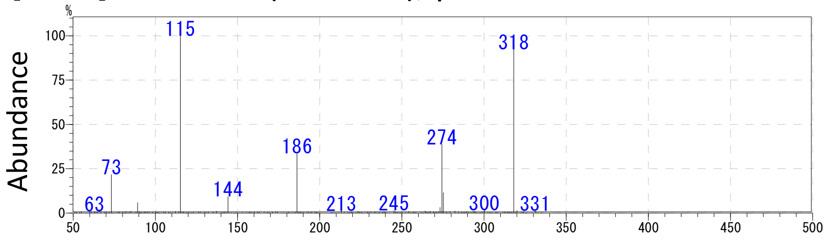

[4-^{13}C]malic acid (3TBDMS), precursor ion: *m/z* = 318

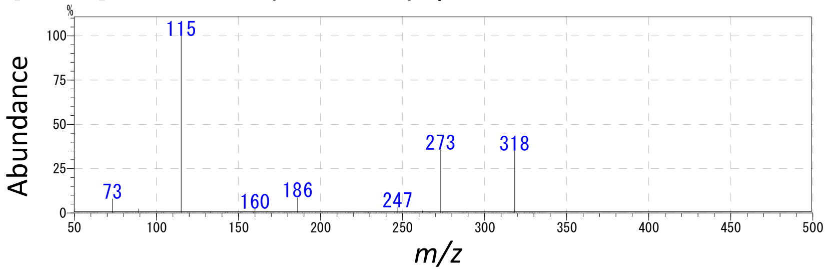

[U-^{13}C]malic acid (3TBDMS), precursor ion: *m/z* = 320

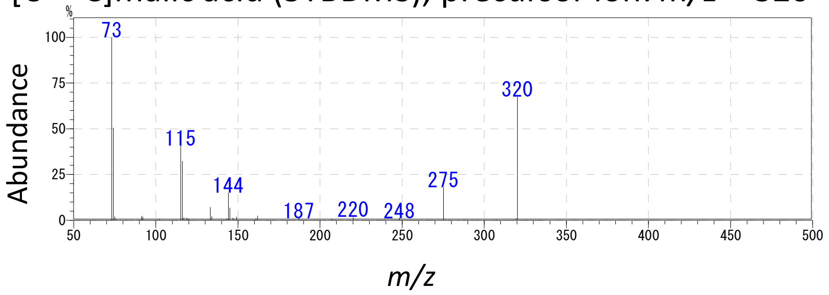

Supplement: Supplementary Data [file massspectrometry-8-1-A0073-s001.pdf]
